# IL-1 receptor antagonist-deficient mice develop autoimmune arthritis due to intrinsic activation of IL-17-producing CCR2^+^Vγ6^+^γδ T cells

**DOI:** 10.1038/ncomms8464

**Published:** 2015-06-25

**Authors:** Aoi Akitsu, Harumichi Ishigame, Shigeru Kakuta, Soo-hyun Chung, Satoshi Ikeda, Kenji Shimizu, Sachiko Kubo, Yang Liu, Masayuki Umemura, Goro Matsuzaki, Yasunobu Yoshikai, Shinobu Saijo, Yoichiro Iwakura

**Affiliations:** 1 Laboratory of Molecular Pathogenesis, Center for Experimental Medicine and Systems Biology, The Institute of Medical Science, The University of Tokyo, Tokyo 108-8639, Japan; 2 Department of Biophysics and Biochemistry, Graduate School of Science, The University of Tokyo, Tokyo 113-0032, Japan; 3 Research Fellow of the Japan Society for the Promotion of Science (JSPS), Tokyo 102-0083, Japan; 4 Core Research for Evolutional Science and Technology (CREST), Japan Science and Technology Agency, Saitama 332-0012, Japan; 5 Division of Experimental Animal Immunology, Center for Animal Disease Models, Research Institute for Biomedical Sciences, Tokyo University of Science, Chiba 278-0022, Japan; 6 Tropical Biosphere Research Center, University of the Ryukyus, Okinawa 903-0213, Japan; 7 Research Center for Prevention of Infectious Diseases, Medical Institute of Bioregulation, Kyushu University, Fukuoka 812-8582, Japan

## Abstract

Interleukin-17 (IL-17)-producing γδ T (γδ17) cells have been implicated in inflammatory diseases, but the underlying pathogenic mechanisms remain unclear. Here, we show that both CD4^+^ and γδ17 cells are required for the development of autoimmune arthritis in IL-1 receptor antagonist (IL-1Ra)-deficient mice. Specifically, activated CD4^+^ T cells direct γδ T-cell infiltration by inducing CCL2 expression in joints. Furthermore, IL-17 reporter mice reveal that the Vγ6^+^ subset of CCR2^+^ γδ T cells preferentially produces IL-17 in inflamed joints. Importantly, because IL-1Ra normally suppresses IL-1R expression on γδ T cells, IL-1Ra-deficient mice exhibit elevated IL-1R expression on Vγ6^+^ cells, which play a critical role in inducing them to produce IL-17. Our findings demonstrate a pathogenic mechanism in which adaptive and innate immunity induce an autoimmune disease in a coordinated manner.

## Abstract (web summary)

Control of γδ T-cell activation remains incompletely understood. Here the authors show that during autoimmune arthritis development αβ CD4^+^ T cells recruit a subset of IL-17-producing γδ T cells to the joints, and that both components are essential to cause pathology in a mouse model of the disease.

## Graphical abstract

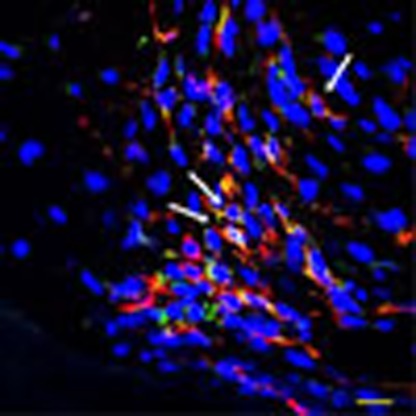

Interleukin (IL)-17 plays important roles in the development of autoimmune diseases, such as rheumatoid arthritis and psoriasis, by inducing expression of proinflammatory cytokines and chemokines, recruiting neutrophils and activating T cells and B cells^1^,^2^. Although helper CD4^+^ T (Th17) cells are well-known producers of IL-17 that contribute to the development of autoimmune diseases, recent studies showed that innate immune cells and innate-like cells are also important sources of IL-17 in local inflammatory tissues^3^,^4^. Mouse autoimmune disease models have revealed that IL-17-producing γδ T (γδ17) cells are an important innate source of IL-17 (refs 5, 6, 7, 8, 9, 10, 11, 12, 13). In collagen-induced arthritis, experimental autoimmune encephalomyelitis and psoriasis-like skin inflammation, the synergy between γδ17 and αβ T cells is important for disease development^5^,^6^,^11^,^14^, but it remains unclear how γδ17 cells induce tissue-specific inflammation.

γδ17 cells share many characteristics with Th17 cells. However, in contrast to Th17 cells in which differentiation in the periphery is required for IL-17 production, the functional potential of γδ17 cells is already determined during intrathymic development^15^,^16^,^17^. These γδ thymocytes, which express the transcription factor RORγt and the signature cytokine receptor IL-23R^18^, leave the thymus as functionally committed cells^19^. Therefore, γδ T cells produce IL-17 directly following stimulation with IL-1β and IL-23 without T cell receptor (TCR) stimulation in the periphery^5^,^13^. Although the expression of IL-23R on γδ17 cells is constitutive^5^, expression of IL-1R in the periphery is tissue-type dependent^20^. *Il1r1*^−/−^ mice or anti-IL-1R monoclonal antibody (mAb) treatment abrogates IL-17 production in γδ T cells^11^,^20^, suggesting that IL-1R expression plays a critical role in IL-17 production. However, the regulatory mechanism of IL-1R expression remains unclear. In addition to RORγt, transcription factors such as Blk^21^, Hes-1 (ref. 22), NFκB^23^, SOX4 and SOX13 (ref. 24) are also required for γδ17 cell development.

In mice, the TCRγ locus consists of seven *Vγ* (*Vγ1*–*Vγ7*) genes that are closely correlated with effector functions, although in most strains Vγ3 is a pseudogene^25^. IL-17 is produced by Vγ4^+^ and Vγ6^+^ γδ T cells^26^ (Heilig and Tonegawa's nomenclature^27^). Although overall gene expression patterns are similar between these two subsets^18^, each subset has distinct features. Vγ6^+^ γδ T cells express the invariant Vγ6/Vδ1 TCR, develop only in the late-embryonic thymus and preferentially localize in the uterus, vagina, lung, dermis and peritoneal cavity^28^,^29^. On the other hand, Vγ4^+^ γδ T cells have a more diverse TCR repertoire, and develop in both foetal and adult thymus. Subsequently, they circulate in blood and reside in the dermis and secondary lymphoid organs^30^. However, the differences between the pathogenic roles of Vγ6^+^ and Vγ4^+^ γδ17 cells, particularly the contribution of Vγ6^+^ γδ17 cells to inflammatory diseases, remain unclear.

The mechanism of γδ17 cell migration into inflammatory sites is also poorly understood. Recent gene array analysis showed that the expression of chemokine receptors such as CCR6, CCR2 and CXCR6 is upregulated in γδ17 thymocytes^18^. Moreover, CCR6 expression is often used as a marker of γδ17 cells^17^. However, it remains unknown whether these chemokine receptors function in γδ17 cell migration.

IL-1 receptor antagonist (IL-1Ra, gene symbol: *Il1rn*) is an endogenous inhibitor of IL-1 activity that competes with IL-1α and IL-1β for IL-1R binding. IL-1Ra-deficient (*Il1rn*^−/−^) mice spontaneously develop arthritis in an IL-17- and T-cell-dependent manner^31^,^32^,^33^, suggesting that excess IL-1 signalling caused by IL-1Ra deficiency induces IL-17 production from T cells and the development of arthritis. Here, we found that γδ17 cells are the main producers of IL-17 in joints of *Il1rn*^−/−^ mice, and that recruitment of CCR2^+^ γδ T cells to the joints via induction of CCL2 by CD4^+^ T cells is important for the development of arthritis. Both Vγ6^+^ and Vγ4^+^ γδ T cells were recruited to the joints, but only the Vγ6^+^ subset efficiently produced IL-17, because *Il1rn*^−/−^ Vγ6^+^ cells intrinsically expressed high levels of IL-1R due to the loss of downregulation of IL-1R expression by IL-1Ra. These observations provide novel insights into the pathogenic mechanism underlying the development of autoimmune arthritis in *Il1rn*^−/−^ mice.

## Results

### γδ T cells mainly produce IL-17 in *Il1rn* ^−/−^ mouse joints

**Figure 1:**
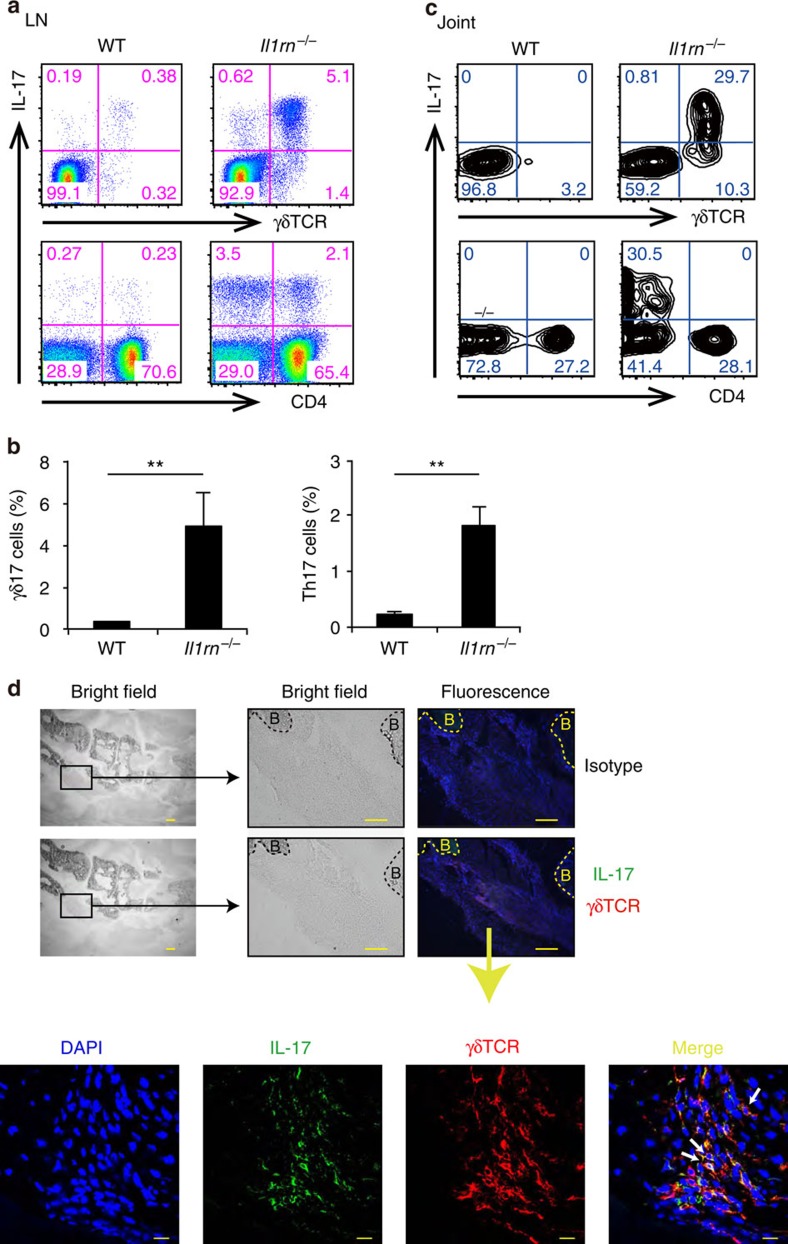
γδ T cells are the main source of IL-17 in the inflamed joints of *Il1rn*^−/−^ mice. (**a–c**) Flow cytometry of LN cells from WT (*n*=3) and arthritic *Il1rn*^−/−^ mice (*n*=3) (**a**) or joint-infiltrating cells from WT (pool of five mice) and arthritic *Il1rn*^−/−^ mice (pool of two mice) (**c**). All cells were stimulated with P/I for 5 h, and then stained for intracellular IL-17. Numbers refer to percent cells in CD3ɛ^+^ cells. Quantification of IL-17^+^ γδ TCR^+^ and IL-17^+^ CD4^+^ in CD3ɛ^+^ cells are shown (**b**). **P<0.01 versus WT mice (unpaired Student's *t*-test). Data show mean±s.e.m. (**d**) Frozen sections of arthritic joints of *Il1rn*^−/−^ mice were co-stained with anti-IL-17 Ab (green), anti-γδ TCR Ab (red) and 4,6-diamidino-2-phenylindole (DAPI) (blue). Sections were observed under a fluorescence microscope (top and middle panels, scale bars, 100 μm), and a confocal microscope (bottom panels, scale bars, 10 μm). B, bone. IL-17^+^ γδ TCR^+^ cells are shown by white arrows. Similar results were obtained in six other *Il1rn*^−/−^ mice. All data except **d** are representative of >5 independent experiments.

We analysed IL-17-producing cells in arthritic *Il1rn*^−/−^ mice. Both γδ17 cell and Th17 cell populations were elevated in draining lymph nodes (LNs) of affected joints (Fig. 1a,b), whereas proportions of IL-17^+^CD8^+^ T cells and IL-17^+^DX5^+^ T cells were unchanged (Supplementary Fig. 1). Notably, most joint-infiltrating IL-17-producing cells were γδ T cells, whereas Th17 cells were rare (Fig. 1c). Moreover, immunofluorescence staining revealed that IL-17 in the joints was primarily expressed in γδ T cells (Fig. 1d). These observations suggest that γδ17 cells play a pathogenic role in the development of arthritis in *Il1rn*^−/−^ mice.

### γδ and CD4^+^ T cells are involved in arthritis development

**Figure 2:**
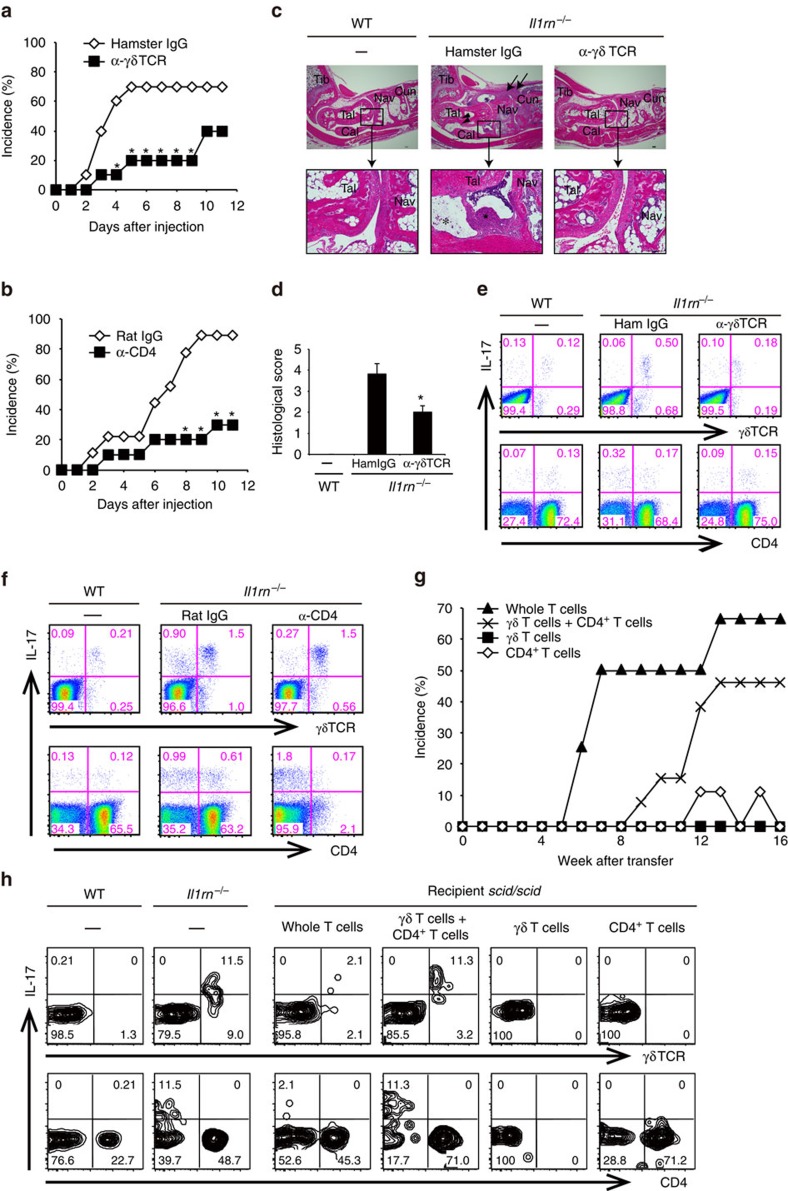
Collaboration between CD4^+^ T cells and γδ17 cells is important for the development of arthritis. (**a**,**b**) Suppression of arthritis development in *Il1rn*^−/−^ mice after treatment with anti-γδ TCR (**a**) or anti-CD4 (**b**) mAb. Non-arthritic *Il1rn*^−/−^ mice at the age of 4 weeks were injected on days 0, 3, 7 and 10 (ages of 28, 31, 35 and 38 days) with anti-γδ TCR mAb (▪, *n*=10) or isotype-matched hamster IgG (◇, *n*=10) (**a**), or with anti-CD4 mAb (▪, *n*=10) or isotype-matched rat IgG (◇, *n*=9) (**b**). *P<0.05 versus control IgG, assessed by the *χ*^2^-test. (**c**,**d**) Non-arthritic *Il1rn*^−/−^ mice at the age of 20 days were injected with anti-γδ TCR mAb or hamster IgG every 3 days (ages of 20, 23 and 26 days), and mice were killed at the age of 27 days. Representative haematoxylin and eosin-stained sections of ankle joints in non-treated WT mouse (left column, *n*=6) and *Il1rn*^−/−^ mouse treated with control hamster IgG (middle column, *n*=6) or α-γδ TCR mAb (right column, *n*=5) are shown. Synovial cell proliferation and inflammatory cell infiltration (arrows), bone erosion (arrowheads), fibrin clots (*) and pannus formation (★) in control *Il1rn*^−/−^ mouse (middle column) are indicated. Scale bars, 100 μm. Tib, tibia; Tal, talus; Cal, calcaneum; Nav, navicular bone; Cun, cuneiform bone (**c**). (**d**) The means of histological scores are shown. *P<0.05 versus *Il1rn*^−/−^ mice treated with hamster IgG. (**e**,**f**) Flow cytometry of LN cells from antibody-treated *Il1rn*^−/−^ mice. Cells were collected from *Il1rn*^−/−^ mice, 8 days after the first injection with anti-γδ TCR mAb or hamster IgG-treated (**e**) or 11 days after the first treatment with anti-CD4 mAb or rat IgG (**f**) or from non-treated WT mice. Cells were stimulated with P/I for 5 h, and then stained for intracellular IL-17. Numbers refer to percentages in CD3ɛ^+^ cells. (**a–f**) Data are representative of two independent experiments. (**g**,**h**) *scid/scid* mice at the age of 4 weeks were transferred with γδ T cells from *Cd4*^−/−^*Il1rn*^−/−^ mice (▪, *n*=8), CD4^+^ T cells from *Tcrd*^−/−^*Il1rn*^−/−^ mice (◇, *n*=9), Thy1.2^+^ T cells (whole-T cells) from *Il1rn*^−/−^ mice (▪, *n*=8) or γδ T cells from *Cd4*^−/−^*Il1rn*^−/−^ mice plus CD4^+^ T cells from *Tcrd*^−/−^*Il1rn*^−/−^ mice (× , *n*=13). Incidence of arthritis is shown (**g**). Flow cytometry of the joint-infiltrating cells of *scid/scid* mice after 18 weeks of transfer, or age-matched and non-treated WT or *Il1rn*^−/−^ mice (**h**). Cells were stimulated with P/I for 5 h, and then stained for intracellular IL-17. Numbers refer to percentage in CD3ɛ^+^ cells. Data are pooled from **g** or representative of (**h**) two independent experiments.

To analyse the pathogenic roles of γδ T cells and CD4^+^ T cells, we injected either anti-γδ TCR or anti-CD4 mAb into *Il1rn*^−/−^ mice before onset of disease. Both antibody treatments significantly suppressed the incidence of arthritis (Fig. 2a,b). Although the severity scores, determined by the swelling and ankylosing changes of the affected ankles of antibody-treated mice, were similar to those of untreated mice (Supplementary Fig. 2a,b), the microscopic histological scores significantly decreased following treatment with anti-γδ TCR mAb (Fig. 2c,d). Anti-γδ TCR mAb treatment depleted 70–90% of γδ T cells at early time points, but the population gradually recovered (Supplementary Fig. 2c), probably due to the development of antibodies against this mAb. Nonetheless, the anti-γδ TCR mAb treatment greatly decreased the γδ17 population (Fig. 2e; Supplementary Fig. 2d), suggesting that anti-γδ TCR mAb effectively depleted γδ17 cells, at least at earlier times. IL-17 production in the γδ TCR^−^ population was not elevated in these mice (Supplementary Fig. 2d). Most populations of CD4^+^ T cells, including Th17 cells, remained depleted in *Il1rn*^−/−^ mice on day 11 after anti-CD4 mAb treatment (Fig. 2f; Supplementary Fig. 2e). These results suggest that both γδ T cells and CD4^+^ T cells are involved in the development of arthritis in *Il1rn*^−/−^ mice. However, arthritis developed normally in *Tcrd*^−/−^*Il1rn*^−/−^ mice (Supplementary Fig. 2f), and IL-17-producing CD4^−^CD8^−^γδTCR^−^ T cells were increased in the LNs and joints of these mice (Supplementary Fig. 2g).

### Both γδ17 and CD4^+^ T cells collaborate to develop arthritis

Next, we directly examined the pathogenic role of γδ T cells and CD4^+^ T cells by adoptive cell transfer. γδ T cells derived from *Cd4*^−/−^*Il1rn*^−/−^ mice and/or CD4^+^ T cells derived from *Tcrd*^−/−^*Il1rn*^−/−^ mice were transferred into *scid/scid* mice. We found that *scid/scid* mice that received transfer of whole-*Il1rn*^−/−^ T cells or a mixture of γδ and CD4^+^ T cells developed arthritis, whereas those that received transfer of γδ or CD4^+^ T cells alone did not (Fig. 2g). Instead, development of inflammation was observed in other organs, such as the colon and dermis, when γδ T cells alone were transferred (Supplementary Fig. 2h). When total T cells or a mixture of γδ and CD4^+^ T cells were transferred, γδ17 cells were present in arthritic joints (Fig. 2h); however, no γδ T cells were located in the joints when γδ T cells were transferred alone. When CD4^+^ T cells were transferred, no Th17 cells were observed in the joints, although IL-17^−^CD4^+^ T cells were found (Fig. 2h). In contrast to the joints, γδ17 and Th17 cells were observed in LNs of *scid/scid* mice that received transfer of γδ and CD4^+^ T cells, respectively (Supplementary Fig. 2i). Thus, *Il1rn*^−/−^ CD4^+^ T cells are required for the recruitment of γδ T cells to the joints, and γδ T cells are required for the production of IL-17.

### Joint-infiltrating γδ17 cells predominantly express CCR2

To analyse γδ17 cells in joints, we generated an IL-17 reporter (*Il17*^*g/g*^) mouse, in which IRES–eGFP was inserted into the *Il17a* locus without affecting IL-17 production (Supplementary Fig. 3a,b). Similar to *Il1rn*^−/−^ mice, *Il17*^*g/g*^*Il1rn*^−/−^ mice spontaneously developed arthritis (Supplementary Fig. 3c). Green fluorescent protein (GFP) expression correlated with intracellular IL-17 expression in joint-infiltrating cells from *Il17*^*g/g*^*Il1rn*^−/−^ mice after phorbol myristate acetate and ionomycin (P/I) stimulation (Supplementary Fig. 3d), indicating that GFP expression accurately reflects IL-17 expression. Consistent with intracellular IL-17 staining in *Il1rn*^−/−^ mice (Fig. 1c), joint GFP expression was mostly limited to γδ T cells (Supplementary Fig. 3e). GFP expression was detectable without any stimulation in joints, whereas only few were detected in LNs (Supplementary Fig. 3f,d), suggesting that γδ17 cells were activated in joints.

**Figure 3:**
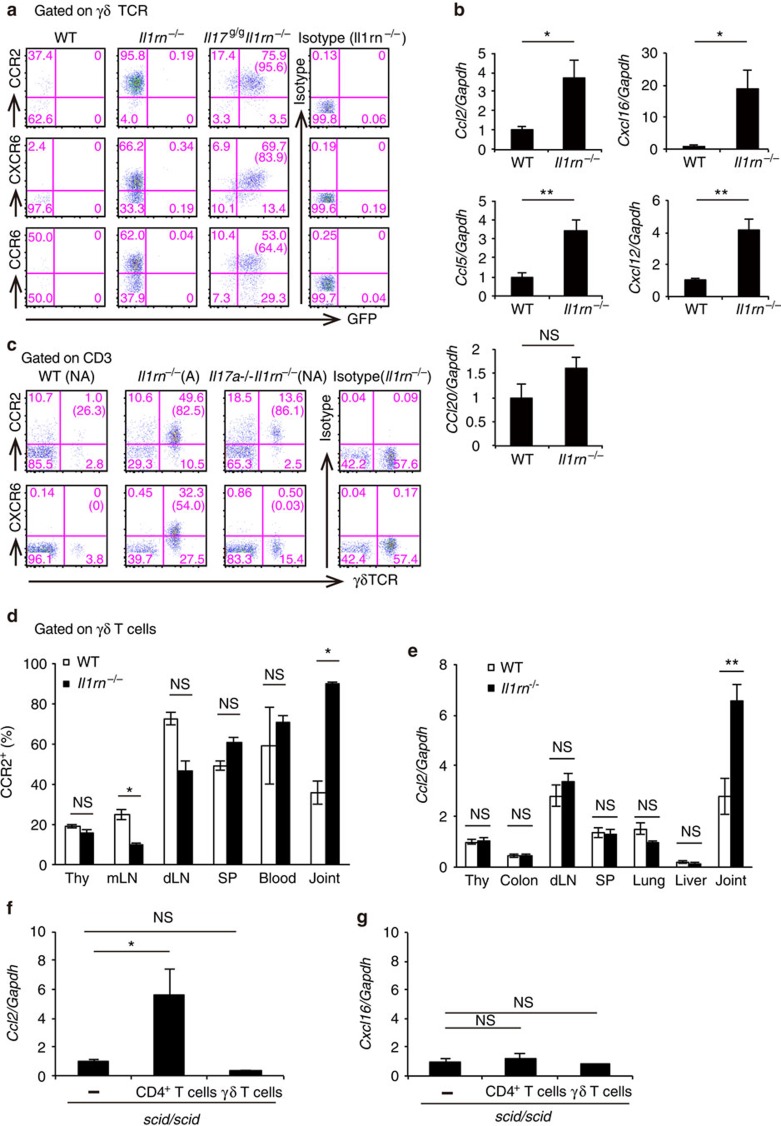
CCR2^+^ γδ17 cells predominantly accumulate in *Il1rn*^−/−^ mouse joints. (**a**) Expression of chemokine receptors and GFP on joint-infiltrating cells from WT (*n*=3, pool of two mice each), *Il1rn*^−/−^ (*n*=3) and *Il17*^*g/g*^*Il1rn*^−/−^ (*n*=3) mice at 16 weeks of age. Numbers represent percentages in CD3ɛ^+^ γδ TCR^+^ cells. Numbers in parentheses represent percentages in GFP^+^ CD3ɛ^+^ γδ TCR^+^ cells. (**b**) Quantitative PCR (qPCR) analysis of the transcripts of CCL2 (*Ccl2*), CXCL16 (*Cxcl16*), CCL20 (*Ccl20*), CCL5 (*Ccl5*) and CXCL12 (*Cxcl12*) in joints of WT (*n*=4) or *Il1rn*^−/−^ (*n*=4) mice. Values are shown relative to those in WT mice. *P<0.05; **P<0.01; NS, not significant (versus WT mice) (unpaired Student's *t*-test). (**c**) Flow cytometry of the joint-infiltrating γδ T cells from WT, arthritic *Il1rn*^−/−^ and non-arthritic *Il17a*^−/−^*Il1rn*^−/−^ mice. Numbers represent percentages in CD3ɛ^+^ cells. Numbers in parentheses represent percentages in CD3ɛ^+^ γδ TCR^+^ cells. (**d**) Contents of CCR2^+^ cells in CD3ɛ^+^ γδ TCR^+^ cells in the thymus (Thy), mesenteric LNs (mLN), draining LNs (dLN), spleens (SP), blood and joints of WT (*n*=3) and *Il1rn*^−/−^ (*n*=3) mice at the age of 14 weeks. *P<0.05; NS (versus WT mice) (unpaired Student's *t*-test). (**e**) Levels of CCL2 mRNA in whole tissue were measured by qPCR in various tissues from WT (*n*=5) and *Il1rn*^−/−^ (*n*=5) mice at the age of 8 weeks. Values are shown relative to those in WT mouse thymus total RNA. **P<0.01; NS (versus WT mice) (unpaired Student's *t*-test). (**f**,**g**) qPCR analysis of mRNA for CCL2 (**f**) and CXCL16 (**g**) in joints of *scid/scid* mice after 24 weeks of transfer of CD4^+^ T cells (*n*=4) or γδ T cells (*n*=3), or age-matched *scid/scid* mice (*n*=3). Values are shown relative to those in control *scid/scid* mice. *P<0.05; NS versus control *scid/scid* mice (unpaired Student's *t*-test). mRNA expression was normalized to that of *Gapdh* (**b**,**e–g**). All data except **a** and **c** show mean±s.e.m. Data are representative of two (**c–g**) or >3 (**a**,**b**) independent experiments.

Several chemokine receptors, including CCR2, CXCR6 and CCR6, were expressed on joint-infiltrating γδ T cells in *Il1rn*^−/−^ mice (Supplementary Fig. 4a). In particular, almost all γδ T cells expressed CCR2, but not CCR5, CXCR4 or CCR9. Using these *Il17*^*g/g*^*Il1rn*^−/−^ mice, we found that about 95% of γδ17 cells expressed CCR2, and that relatively large proportions of γδ17 cells also expressed CXCR6 and CCR6 (Fig. 3a).

Next, we examined chemokine expression in joints. Consistent with our previous microarray analysis^34^, expression of *Ccl2* (ligand for CCR2), *Cxcl16* (ligand for CXCR6), *Ccl5* (ligand for CCR5) and *Cxcl12* (ligand for CXCR4) was significantly elevated in joints of *Il1rn*^−/−^ mice relative to wild-type (WT) mice, whereas expression of *Ccl20* (ligand for CCR6) was unchanged (Fig. 3b). Moreover, we detected moderate levels of γδ T-cell infiltration in the non-arthritic joints of *Il17a*^−/−^*Il1rn*^−/−^ mice^32^. In these mice, γδ T cells expressed high levels of CCR2, but not CXCR6 (Fig. 3c), indicating that the accumulation of CCR2^+^ γδ T cells is not a result of inflammation. These observations suggest that the CCL2–CCR2 axis is involved in the recruitment of γδ T cells into joints.

### *Il1rn* ^−/−^ CD4^+^ cells induce *Ccl2* expression in joints

CCR2^+^ γδ T cells accumulated preferentially in arthritic joints, but not other organs, of *Il1rn*^−/−^ mice (Fig. 3d; Supplementary Fig. 4b). Consistent with this, expression of *Ccl2* (Fig. 3e), but not *Ccl20* (Supplementary Fig. 4c), was elevated in joints of *Il1rn*^−/−^ mice. Moreover, expression of *Ccl2*, but not *Cxcl16*, was specifically elevated in joints of *scid/scid* mice when *Il1rn*^−/−^ CD4^+^ T cells, but not γδ T cells, were transferred (Fig. 3f,g). Thus, activated CD4^+^ T cells induce *Ccl2* expression in synovial resident cells and cause migration of CCR2^+^ γδ T cells.

### CCL2 recruits γδ T cells to joints and induces arthritis

**Figure 4:**
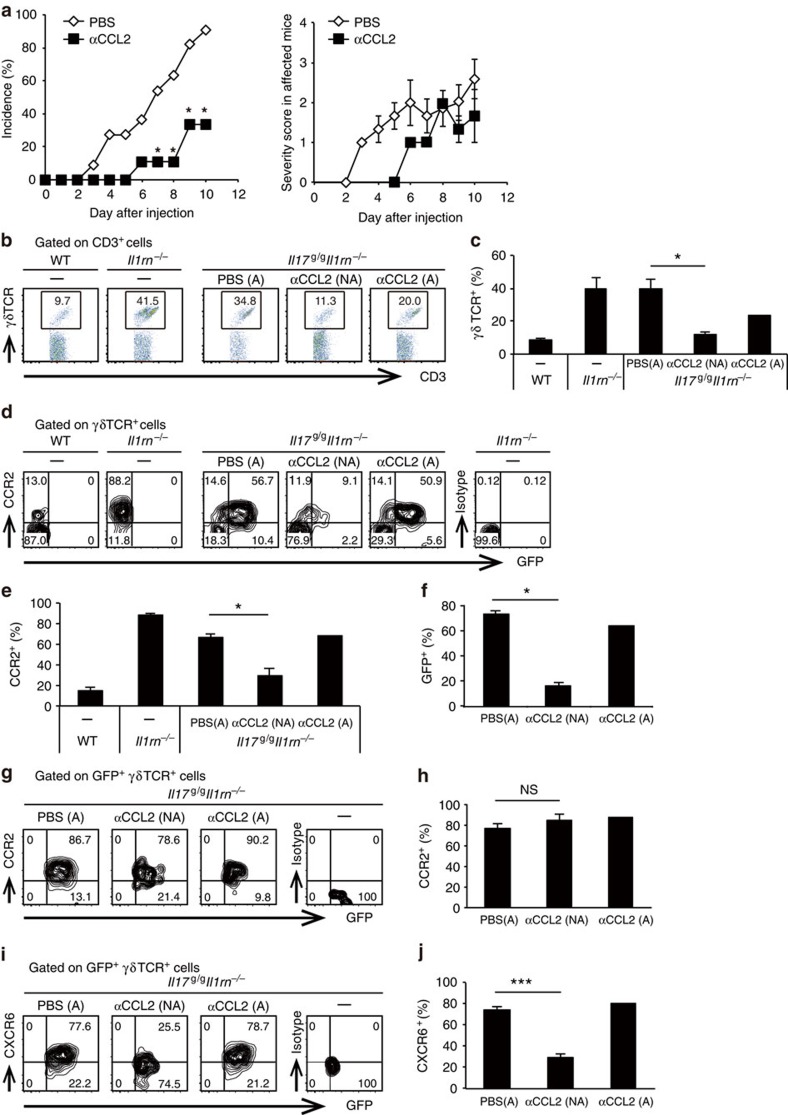
CCL2 is essential for γδ T-cell infiltration into joints and arthritis development. (**a**) Incidence (left) and severity scores of arthritis in affected mice (right) in *Il17*^*g/g*^*Il1rn*^−/−^ mice after anti-CCL2 mAb treatment. Non-arthritic *Il17*^g/g^*Il1rn*^−/−^ mice at the age of 21 days were injected with anti-CCL2 mAb (▪, *n*=9) or PBS (◇, *n*=11) every 3 days (ages of 21, 24, 27 and 30 days). *P<0.05 versus treatment with PBS, assessed by the *χ*^2^-test. Data represent a pool of two independent experiments. (**b–j**) Flow cytometry of joint-infiltrating cells from *Il17*^*g/g*^*Il1rn*^−/−^ mice 11 days after the first injection. Cells were collected from PBS-treated arthritic (A) mice (*n*=5) and anti-CCL2 mAb-treated non-arthritic (NA) (*n*=3) or arthritic (A) (*n*=2) mice. Age-matched and non-treated WT (*n*=3) and arthritic *Il1rn*^−/−^ (*n*=3) mice were used as controls. Numbers refer to percentage in CD3ɛ^+^ cells (**b**), in CD3ɛ^+^ γδ TCR^+^ cells (**d**) or in GFP^+^CD3ɛ^+^γδ TCR^+^ cells (**g**,**i**). The average proportions of γδ T cells in CD3ɛ^+^ cells (**c**), CCR2^+^ (**e**) or GFP^+^ (**f**) cells among γδ TCR^+^ cells, or CCR2^+^ (**h**) or CXCR6^+^ (**j**) cells among GFP^+^γδ TCR^+^ cells, are shown. *P<0.05; ***P<0.001; NS, not significant (versus *Il17*^*g/g*^*Il1rn*^−/−^ mice treated with PBS) (unpaired Student's *t*-test). Data show mean±s.e.m. Data (**b–j**) are representative of two independent experiments.

To determine whether elevated CCL2 expression in joints causes γδ17 cell accumulation and disease development in *Il1rn*^−/−^ mice, we injected anti-CCL2 mAb into *Il17*^*g/g*^*Il1rn*^−/−^ mice and analysed joint-infiltrating γδ T cells. Anti-CCL2 mAb treatment significantly suppressed development of arthritis in *Il17*^*g/g*^*Il1rn*^−/−^ mice (Fig. 4a; Supplementary Fig. 5), suggesting an important role for CCL2 in pathogenesis. The frequencies of γδ T cells and CCR2^+^ γδ T cells were significantly reduced in joints of non-arthritic mice after mAb treatment (Fig. 4b–e), indicating that CCL2 is responsible for recruitment of CCR2^+^ γδ T cells into joints. In mAb-treated non-arthritic mice, only a few GFP^+^ γδ T cells were detected (Fig. 4d,f), suggesting a pathogenic role for joint-infiltrating γδ17 cells in development of arthritis. Notably, when GFP^+^ γδ T cells were gated, CCR2 was expressed at similar levels even in cells from anti-CCL2 mAb-treated non-arthritic mouse joints (Fig. 4g,h), suggesting that CCR2 expression in γδ17 cells is required for the development of arthritis. On the other hand, the correlation between CXCR6 expression and disease development suggests that CXCR6 expression is a result of inflammation (Fig. 4i,j). These data indicate that CCR2^+^ γδ T cells migrate into joints in response to high levels of CCL2 in *Il1rn*^−/−^ mouse joints, leading to development of arthritis.

### IL-1Ra suppresses IL-1R expression on γδ T cells

**Figure 5:**
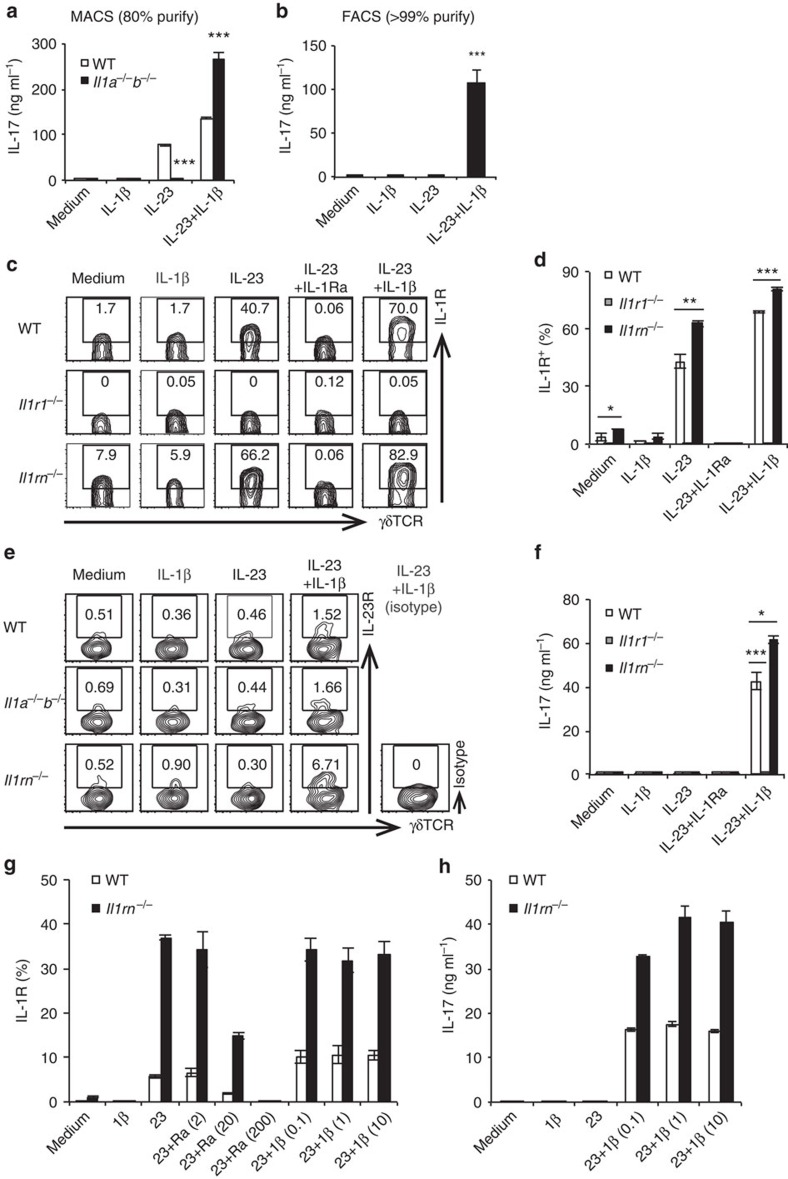
IL-23 induces expression of IL-1R on the surface of γδ T cells, whereas IL-1Ra suppresses its expression. (**a**,**b**) Concentrations of IL-17 in culture supernatants of magnetic-activated cell sorting (MACS)-purified (about 80%) (**a**) or FACS-purified (>99% pure) (**b**) splenic γδ T cells from pools of 16 WT mice (**a**,**b**) or 16 *Il1a*^−/−^*b*^−/−^ mice (**b**) stimulated for 3 days with medium only, IL-1β, IL-23 or IL-23 plus IL-1β, without γδ TCR stimulation. IL-17 was detected by enzyme-linked immunosorbent assay (ELISA). ***P<0.001 versus WT mice (**a**); ***P<0.001 versus medium only (**b**) (unpaired Student's *t*-test). (**c–h**) FACS-purified γδ T cells from pooled spleens of WT, *Il1r1*^−/−^, *Il1a*^−/−^*b*^−/−^ or *Il1rn*^−/−^ mice (11–16 mice each) were stimulated for 3 days with medium only, IL-1β, IL-23, IL-23 plus IL-1β or IL-23 plus IL-Ra. Flow cytometry of γδ T cells stained for surface IL-1R (**c**) and IL-23R (**e**) are shown. Quantification of IL-1R^+^ γδ T cells is indicated in **d** and **g**. Concentrations of IL-17 in culture supernatants were determined by ELISA (**f**,**h**). *P<0.05; **P<0.01; ***P<0.001 (versus WT mice) (unpaired Student's *t*-test). Numbers in parentheses indicate the concentration of cytokines (ng ml^−1^). Representative data (**c**,**e**) and mean±s.e.m. (**a**,**b**,**d**,**f**,**g**,**h**) of triplicate cultures are shown. All data are representative of two or three independent experiments.

Next, we analysed the mechanism of IL-17 induction in γδ T cells. Consistent with a previous report^35^, IL-23 alone or IL-23 plus IL-1β induced IL-17 production from magnetic-activated cell sorting (MACS)-purified splenic γδ T cells (purity: 80%) (Fig. 5a). However, MACS-purified *Il1a*^−/−^*Il1b*^−/−^ γδ T cells or fluorescence-activated cell sorting (FACS)-purified WT γδ T cells (purity: >99%) did not respond to IL-23 alone, and IL-1β was required to induce IL-17 (Fig. 5a,b), suggesting that IL-1β is essential for the IL-17 induction in γδ T cells. Consistent with these results, the development of arthritis was completely suppressed in *Il1b*^−/−^*Il1rn*^−/−^ mice (Supplementary Table 1).

We then analysed the effects of IL-1β and IL-23 on expression of genes encoding transcription factors characteristic of Th17 cells, such as RORγt, RORα, IκBζ and BATF. RORγt expression in γδ T cells was drastically increased by addition of IL-1β and IL-23 together, but only marginally by IL-1β or IL-23 alone (Supplementary Fig. 6a). A similar effect was observed on RORα expression. IκBζ and BATF were induced by IL-1β alone, and the increase in their expression was synergistically enhanced by the addition of IL-23.

IL-23 remarkably increased IL-1R expression on γδ T cells (Fig. 5c,d; Supplementary Fig. 6b). On the other hand, IL-23R was expressed on unstimulated γδ T cells from WT, *Il1a*^−/−^*b*^−/−^ and *Il1rn*^−/−^ mice, and its expression was not enhanced by the addition of IL-1β (Fig. 5e). IL-1β or IL-23 signalling alone was not sufficient for induction of IL-17; instead, synergistic activation by IL-1β and IL-23 was required (Fig. 5f; Supplementary Fig. 6c).

IL-1R expression induced by IL-23 was suppressed by the addition of exogenous IL-1Ra, and IL-1R expression was elevated on *Il1rn*^−/−^ γδ T cells (Fig. 5c,d), indicating that IL-23 and IL-1Ra reciprocally regulates IL-17 production by regulating IL-1R expression. Consistent with the elevation in IL-1R expression, IL-17 production in response to IL-23 and IL-1β stimulation was higher in *Il1rn*^−/−^ γδ T cells than in WT γδ T cells (Fig. 5f; Supplementary Fig. 6c). *Il1rn*^−/−^ γδ T cells were less sensitive than WT γδ T cells to inhibition of IL-1R expression by IL-1Ra, and the maximum levels of IL-1R expression induced by IL-1β and IL-23 on WT γδ T cells were lower than those on *Il1rn*^−/−^ γδ T cells (Fig. 5g). These results indicate that IL-1R expression is abnormally elevated in *Il1rn*^−/−^ γδ T cells, resulting in higher IL-17 production in these cells (Fig. 5h). Thus, these results suggest that IL-1Ra is not merely an antagonist of IL-1R for the binding of IL-1α and IL-1β, but is also an important regulator of IL-1R expression on the cell surface.

### Vγ6^+^ subset is the main IL-17 producer in *Il1rn* ^−/−^ joints

To identify the γδ subset responsible for IL-17 production in *Il1rn*^−/−^ mouse joints, we examined the Vγ subset in joint-infiltrating γδ T cells. Proportions of Vγ subsets were analysed using Vγ TCR-specific antibodies except for Vγ6, because no anti-Vγ6 antibody was available. We found that γδ T cells in the joints consisted of only two major populations: Vγ4^+^ and an ‘other' population that was stained by none of antibodies against Vγ1, Vγ2, Vγ4, Vγ5 or Vγ7 TCR (Supplementary Fig. 7a). We assumed this ‘other' Vγ subset represented Vγ6^+^ cells, because only Vγ6^+^ cells are predicted to be unstained by all of these antibodies. γδ T-cell subsets in LNs were heterogeneous, but only the Vγ4^+^ and putative Vγ6^+^ subsets were capable of producing IL-17 (Supplementary Fig. 7b).

**Figure 6:**
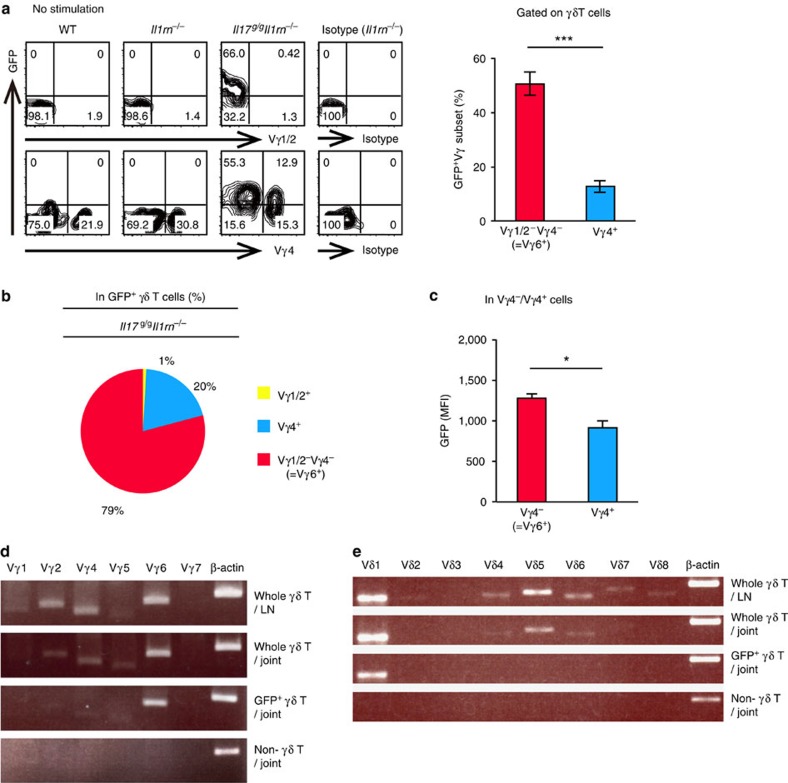
The Vγ6^+^ γδ T-cell subset is the major source of IL-17 in *Il1rn*^−/−^ mouse joints. (**a–c**) GFP expression in joint-infiltrating γδ T cells from WT, *Il1rn*^−/−^ and *Il17*^*g/g*^*Il1rn*^−/−^ mice at 16 weeks of age. Numbers refer to percentage in CD3ɛ^+^ γδ TCR^+^ cells (**a**, left). Quantification of GFP^+^ Vγ subsets in CD3ɛ^+^ γδTCR^+^ cells is shown (**a**, right). Numbers in the pie chart show the percentages of the indicated Vγ subset among GFP^+^ γδ TCR^+^ CD3ɛ^+^ cells, and represent the average of three mice (**b**). Mean fluorescence intensity of GFP in Vγ6^+^ (Vγ4^−^) or Vγ4^+^ cells is shown in **c**. *P<0.05, ***P<0.001 (versus Vγ4^+^ cells) (unpaired Student's *t*-test). Data show the mean±s.e.m. of three mice. (**d**,**e**) Vγ (**d**) and Vδ (**e**) gene expression in γδ T cells. CD3ɛ^+^ γδ TCR^+^ (whole γδ T) cells from LNs (first row) or from joints (second row) of *Il1rn*^−/−^ mice and GFP^+^ CD3ɛ^+^ γδ TCR^+^ (GFP^+^ γδ T) (third row) or CD3ɛ^+^ γδ TCR^−^ (non-γδ T) (fourth row) cells from joints of *Il17*^g/g^*Il1rn*^−/−^ mice, were sorted on a FACSAria, and Vγ and Vδ gene expression was analysed by RT–PCR.

Next, we analysed Vγ subsets in γδ17 cells using *Il17*^*g/g*^*Il1rn*^−/−^ mice. An average of 80% of joint-infiltrating GFP^+^ γδ T cells were unstained by anti-Vγ4 and anti-Vγ1/2 antibodies (putative Vγ6^+^ cells), and only 20% of the population was Vγ4^+^ (Fig. 6a,b). Furthermore, mean fluorescence intensity of GFP was significantly higher in Vγ4^−^ cells than in Vγ4^+^ cells (Fig. 6c). Furthermore, the putative Vγ6^+^ cell population of IL-17^+^ γδ T cells (63%) was larger than the Vγ4^+^ cell population (37%), as estimated by intracellular IL-17 staining (Supplementary Fig. 7c). The proportions of Vγ4^+^ cells in IL-17^+^ and in GFP^+^ γδ T cells were slightly elevated following P/I stimulation (Supplementary Fig. 7c,d), probably because GFP as well as IL-17 expression is elevated after P/I stimulation or cell activation^23^,^36^,^37^, and most Vγ6^+^ cells were already activated in *Il1rn*^−/−^ γδ T cells *in vivo*.

We also examined Vγ subset composition by measuring Vγ messenger RNA (mRNA) expression using reverse transcription (RT)–PCR. Vγ6 mRNA was preferentially expressed in FACS-sorted GFP^+^ γδ T cells in joints from *Il17*^*g/g*^*Il1rn*^−/−^ mice, whereas other Vγ mRNAs were also detected in LN γδ T cells (Fig. 6d), consistent with the results of FACS analyses. Vδ1 mRNA was exclusively detected in GFP^+^ γδ T cells in joints (Fig. 6e), suggesting that these cells are of the canonical Vγ6/Vδ1 γδ T-cell subset^38^. Thus, Vγ6^+^ γδ T cells are the major producers of IL-17 in joints of *Il1rn*^−/−^ mice.

### *Il1rn* ^−/−^ Vγ6^+^ cells highly express IL-1R intrinsically

**Figure 7:**
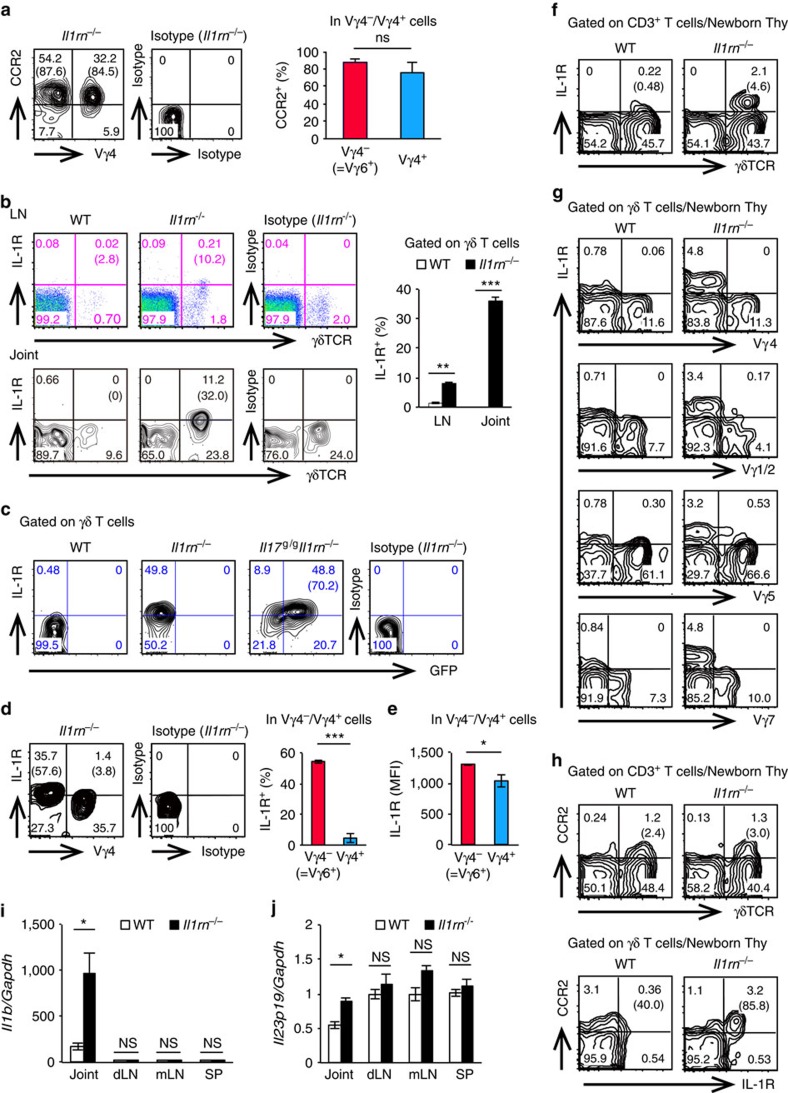
IL-17-producing *Il1rn*^−/−^ Vγ6^+^ γδ T cells intrinsically express high levels of IL-1R. (**a**) Flow cytometry of joint-infiltrating cells from *Il1rn*^−/−^ mice (*n*=3) for the expression of CCR2 and Vγ4. Numbers refer to percentage in CD3ɛ^+^ γδ TCR^+^ cells. Numbers in parentheses represent percentage in Vγ6^+^ (Vγ4^−^) or Vγ4^+^ cells (left). Quantification of CCR2^+^ cells in Vγ6^+^ (Vγ4^−^) or Vγ4^+^ cells is shown (right). NS, not significant versus Vγ4^+^ cells (unpaired Student's *t*-test). (**b**) Flow cytometry of IL-1R expression in LNs and joint-infiltrating cells from WT or *Il1rn*^−/−^ mice. Numbers refer to percentages in CD3ɛ^+^ cells. Numbers in parenthesis represent percentages in CD3ɛ^+^ γδ TCR^+^ cells (left). The percentage of IL-1R^+^ cells among CD3ɛ^+^ γδ TCR^+^ cells is indicated on the right. **P<0.01; ***P<0.001 (versus WT mice) (unpaired Student's *t*-test). (**c**) FACS analysis of IL-1R and GFP expression on joint-infiltrating CD3ɛ^+^ γδ TCR^+^ cells from WT, *Il1rn*^−/−^ and *Il17*^*g/g*^ *Il1rn*^−/−^ mice. Numbers in parentheses represent percentage in GFP^+^ γδ TCR^+^ cells. (**d**,**e**) Flow cytometry of IL-1R expression in joint-infiltrating cells from *Il1rn*^−/−^ mice. Numbers refer to percentages in CD3ɛ^+^ γδ TCR^+^ cells. Numbers in parentheses represent percentages in Vγ6^+^ (Vγ4^−^) or Vγ4^+^ cells (**d**, left). Quantification of IL-1R^+^ cells (**d**, right) and mean fluorescence intensity of IL-1R in Vγ6^+^ or Vγ4^+^ cells are indicated (**e**). *P<0.05; ***P<0.001 (versus Vγ4^+^cells) (unpaired Student's *t*-test). (**f–h**) Flow cytometry of IL-1R expression in γδ T cells in newborn thymus (within the first day after birth) from WT and *Il1rn*^−/−^ mice. IL-1R expression on γδ T cells (**f**) and on different Vγ subsets (**g**), and CCR2 and IL-1R expression on γδ T cells (**h**) are shown. Numbers refer to percentage in CD3ɛ^+^ cells (**f**,**h**, top), or in CD3ɛ^+^ γδ TCR^+^ cells (**g**,**h**, bottom). Numbers in parentheses represent percentage in γδ TCR^+^ cells (**f**,**h**, top) or in IL-1R^+^ γδ TCR^+^ cells (**h**, bottom). (**i**,**j**) qPCR analysis of the transcripts for IL-1β (*Il1b*) (**i**) or IL-23 (*Il23p19*) (**j**) in cells from joints, draining LNs (dLN), mesenteric LNs (mLN) and spleens (SP) from WT or *Il1rn*^−/−^ mice. mRNA levels were normalized against *Gapdh*, and values are shown relative to mLN of WT mice. *P<0.05; NS, not significant (versus WT mice) (unpaired Student's *t*-test). Data show mean±s.e.m. All data are representative of two or three independent experiments.

Because both Vγ6^+^ and Vγ4^+^ cells were present in joints with high levels of CCR2 expression on the surface (Fig. 7a), we asked why IL-17 was preferentially produced by Vγ6^+^ cells in *Il1rn*^−/−^ mouse joints. IL-1R expression on γδ T cells was greatly elevated in both LNs and joints of *Il1rn*^−/−^ mice (Fig. 7b), and most GFP^+^ γδ T cells in *Il117*^*g/g*^*Il1rn*^−/−^ mouse joints were IL-1R^+^ (Fig. 7c). This IL-1R^+^ population was mostly Vγ6^+^ (Fig. 7d). The mean fluorescence intensity of IL-1R was also significantly higher in Vγ6^+^ cells than in Vγ4^+^ cells (Fig. 7e). This elevated IL-1R expression was already present in newborn thymic γδ T cells from *Il1rn*^−/−^ mice (Fig. 7f), and was detected on Vγ6^+^ (Vγ4^−^, Vγ1^/^2^−^, Vγ5^−^ and Vγ7^−^) cells (Fig. 7g). Notably, IL-1R expression on γδ T cells in newborn thymus correlated with CCR2 expression (Fig. 7h). IL-1β mRNA expression was observed even in joints of WT mice, and was greatly elevated in joints of *Il1rn*^−/−^ mice (Fig. 7i). IL-23 mRNA expression in joints was also significantly higher in *Il1rn*^−/−^ mice than in WT mice (Fig. 7j). These observations suggest that joint-infiltrating CCR2^+^Vγ6^+^ *Il1rn*^−/−^ γδ T cells, which intrinsically express high levels of IL-1R, preferentially produce IL-17 in response to IL-1β and IL-23, resulting in development of arthritis.

## Discussion

Here we showed that both CD4^+^ T cells and γδ17 cells are important for development of arthritis in *Il1rn*^−/−^ mice, because antibody-mediated depletion of either γδ T or CD4^+^ T cells suppressed the development of arthritis. Furthermore, upon adoptive transfer, only a mixture of γδ T and CD4^+^ T cells induced arthritis in *scid/scid* mice. γδ17 cells localized in joints of *scid/scid* mice when γδ T cells were transferred along with CD4^+^ T cells, whereas γδ T cells were not detected in joints when γδ T cells were transferred alone. These observations suggest that γδ T cells alone cannot distribute into joints, and CD4^+^ T cells are required for the localization of γδ T cells.

Anti-γδ TCR mAb injection significantly suppressed not only the incidence of arthritis but also the histological severity score, indicating that γδ T cells are involved in development of arthritis. It was recently reported that treatment with anti-γδ TCR mAb results in internalization of γδ TCR rather than γδ T-cell depletion^39^. In our hands, however, the γδ T-cell population was greatly diminished by treatment with this antibody, indicating that the γδ17 population is actually depleted by this antibody. Importantly, the γδ17 population was significantly reduced without a compensatory increase in IL-17^+^ cells in the γδ TCR^–^ population, suggesting that the reduction of γδ17 cells was not the result of replacement of γδ17 cells by hypothetical ‘invisible γδ T cells'^39^. A reduction in IL-17 production following anti-γδ TCR mAb treatment was also reported by another group^40^. Although we examined the effect of γδ T-cell deficiency using *Tcrd*^−/−^ mice, we could not find any effect of this gene mutation on the development of arthritis. Interestingly, we found IL-17-producing CD4^−^CD8^−^γδTCR^−^ T cells were increased in the joints of *Tcrd*^−/−^*Il1rn*^−/−^ mice, suggesting that these cells may substitute for the deficiency of γδ T cells to produce IL-17. We are now further characterizing these cells.

In *Il17*^*g/g*^*Il1rn*^−/−^ mice, most of the joint-infiltrating γδ17 cells expressed CCR2, in association with elevated expression of CCL2 in the joints. Antibody-mediated blockade of CCL2 in *Il17*^*g/g*^*Il1rn*^−/−^ mice reduced infiltration of CCR2^+^ GFP^+^ γδ T cells in joints and suppressed the development of arthritis, suggesting that CCR2^+^ γδ17 cell accumulation in the joints, which is critical for the development of arthritis, is mediated by the CCL2–CCR2 interaction. The CCL2–CCR2 axis-mediated γδ T-cell migration has also been reported as a protection mechanism against tumours^41^. Regarding CCR2 expression, a subset of γδ T cells acquires effector functions and expresses IL-17 during intrathymic development^15^,^16^, and these thymic γδ17 subsets already express some chemokine receptors such as CCR2, CCR6 and CXCR6 (refs 17, 18). In contrast to γδ17 cells in *Il1rn*^−/−^ mice, pathogenic Th17 cells expressing CCR6 are recruited to inflammatory sites, such as joints of SKG mice^42^ and the central nervous system of an experimental autoimmune encephalomyelitis model^43^,^44^, via the CCR6–CCL20 interaction. Because we did not observe a significant increase in CCL20 expression in joints of *Il1rn*^−/−^ mice relative to WT mice, the CCR6–CCL20 axis may not be important for the γδ17 cell migration into joints, even though >50% of joint-infiltrating GFP^+^ γδ T cells in *Il17*^*g/g*^*Il1rn*^−/−^ mice also expressed CCR6. CXCR6 was also expressed on joint-infiltrating γδ17 cells in *Il17*^*g/g*^*Il1rn*^−/−^ mice. However, because this chemokine receptor was not expressed on joint-infiltrating γδ T cells in non-arthritic *Il17a*^−/−^*Il1rn*^−/−^ mice, CXCR6 may not be involved in recruitment of γδ T cells, at least under non-inflammatory conditions.

Importantly, *Il1rn*^−/−^ CD4^+^ T-cell transfer induced CCL2 expression in joints of recipient *scid/scid* mice, suggesting that CD4^+^ T cells directed the migration of CCR2^+^ γδ T cells. We rarely detected Th17 cells in the inflamed joints, indicating that IL-17 production from CD4^+^ T cells is not required for pathogenesis in *Il1rn*^−/−^ mice, at least for induction of local inflammation. Therefore, our results show that CD4^+^ T cells direct the tissue specificity of inflammation, and γδ17 cell-derived IL-17 elicits local inflammation and arthritis in *Il1rn*^−/−^ mice. Although the importance of γδ17 cells in the development of arthritis in the collagen-induced arthritis model was already suggested by Ito *et al*.^13^, the mechanism how these γδ T cells are distributed to joints has not been elucidated. In this report, we first clarified the mechanism how γδ T cells are recruited to the inflammatory site. Actually, when γδ T cells alone were transferred to *scid/scid* mice, development of inflammation was observed in other organs, such as the colon and dermis, suggesting the importance of CD4^+^ T cells for the tissue-specific distribution of γδ T cells. We showed previously that T cells from *Il1rn*^−/−^ mice are hyper-reactive due to the overexpression of CD40L and OX40 on the cell surface, and consequently lose tolerance against self-antigens^32^,^33^.

A combination of IL-1β and IL-23, but not IL-1β or IL-23 alone, induces IL-17 in γδ T cells without TCR engagement^11^,^13^,^20^,^35^. In this report, we showed that IL-23 is required for the induction of IL-1R on γδ T cells, and IL-1β is essential for the induction of IL-17. However, IL-1β alone could not induce IL-17 production in *Il1rn*^−/−^ γδ T cells, even though these cells expressed IL-1R, consistent with a report that IL-1β alone does not induce IL-17 in peritoneum- and lung-derived γδ T cells expressing high levels of IL-1R^20^. These observations suggest that IL-23 plays roles, other than upregulating IL-1R, in the induction of IL-17 expression in γδ T cells. In this context, expression of IL-17 signature transcription factors such as *Rorc*, *Rora*, *Nfkbz* and *Batf* was increased by IL-1β and IL-23 together.

Moreover, IL-23-induced IL-1R expression on γδ T cells was suppressed by exogenous IL-1Ra, suggesting that IL-1Ra not only antagonizes IL-1α and IL-1β for IL-1R binding, but also regulates cell-surface expression of IL-1R. Consistent with this, IL-1R expression on γδ T cells was intrinsically augmented in *Il1rn*^−/−^ mice, making IL-17 production by these *Il1rn*^−/−^ γδ T cells hyper-sensitive to the action of IL-1β and IL-23. *Il1rn*^−/−^ γδ T cells induced arthritis upon transfer into *scid/scid* mice. Because IL-1R expression levels induced by IL-1β and IL-23 were much higher on *Il1rn*^−/−^ γδ T cells than on WT γδ T cells, endogenous low levels of IL-1Ra in the recipients could not suppress IL-1R expression on *Il1rn*^−/−^ γδ T cells. Thus, the strict control of IL-1R expression by IL-1Ra is important for regulation of IL-17 production in γδ T cells.

Children with homozygous point mutations or deletions of the *IL1RN* gene develop life-threatening severe inflammatory diseases with prominent involvement of bone and skin after birth^45^,^46^. The population of IL-17-producing cells is markedly elevated in inflamed skin, and a large amount of IL-1β is produced in mononuclear cells, as in *Il1rn*^−/−^ mice^45^,^46^. Although the pathogenic roles of IL-17 in local inflammation in these patients remain unknown, γδ T cells may be involved in the inflammation. Our observation that exogenous IL-Ra suppresses IL-1R expression on γδ T cells may partly explain why treatment with the recombinant IL-1Ra anakinra completely resolves symptoms in these affected children^45^,^46^.

Using *Il17*^*g/g*^ mice, we showed that IL-17 was preferentially produced in Vγ6^+^ γδ T cells of *Il1rn*^−/−^ mouse joints, although both CCR2^+^ Vγ6^+^ cells and CCR2^+^ Vγ4^+^ cells were localized in the joints. These results suggest that IL-17 production capacity differs in these two γδ T-cell subsets. We found that IL-1R expression in joints is much higher on Vγ6^+^ cells than on Vγ4^+^ cells, explaining why IL-17 is induced preferentially in Vγ6^+^ cells in *Il1rn*^−/−^ mice. This elevated IL-1R expression was observed even in newborn thymus. Notably in this regard, different transcription factors are required for the development of Vγ6^+^ and Vγ4^+^ cell subset^24^, and Vγ6^+^ cells acquire the innate capacity to produce IL-17 in the embryonic thymus^47^. Therefore, during embryonic development, the level of IL-1R is probably intrinsically higher in Vγ6^+^ γδ T cells than in Vγ4^+^ cells.

Vγ6^+^ γδ17 cells are thought to be a tissue-resident, long-lived, self-renewing population^47^. Although our CCL2 inhibition and transfer experiments suggested that LN Vγ6^+^ γδ T cells capable of producing IL-17 were recruited to joints (where they induced arthritis) via CCR2–CCL2 interaction, development of arthritis was not completely suppressed by treatment with anti-CCL2 mAb. Therefore, we could not completely exclude the possibility that these tissue-resident Vγ6^+^ γδ17 cells are also involved in development of arthritis.

γδ17 cells have been observed in the peripheral tissues of several inflammatory diseases^11^,^48^,^49^, and the involvement of γδ17 cells is suggested in some autoimmune models^5^,^7^,^11^,^12^,^13^,^24^. Innate immune cell-derived IL-17 has also been implicated in the pathogenesis of intestinal inflammatory diseases^4^. However, the mechanisms by which these IL-17-producing γδ T cells or innate immune cells are recruited to the inflammatory sites remain largely unknown. In this report, we showed that CD4^+^ T cells determine the tissue specificity, and CD4^+^ T-cell-induced CCL2 recruits CCR2^+^ Vγ6^+^ γδ T cells to the joints. Therefore, our model may explain how IL-17-producing γδ T cells or innate immune cells infiltrate local tissues and induce inflammation in other autoimmune diseases^50^. Taken together, our findings provide important insight into the immunological mechanisms driving tissue-specific autoimmune diseases, and may provide a clue for the development of novel therapies.

## Methods

### Mice

All mice, except C.B.-17 *scid/scid* mice (CLEA Japan, Tokyo, Japan), were on the BALB/cA genetic background. *Il1rn*^−/−^ mice were produced as described^31^,^51^, and backcrossed to BALB/cA (CLEA Japan) for nine generations. *Il17*^*g/g*^ mice were generated as shown in Supplementary Fig. 3a, and backcrossed to BALB/cA mice for eight generations. *Il17*^*g/g*^*Il1rn*^−/−^ mice were produced by crossing *Il17*^*g/g*^ mice with *Il1rn*^−/−^ mice. *Tcrd*^−/−^ mice were generated by Itohara *et al*.^52^. *Cd4*^−/−^ mice were a gift from Dr. Fujii (RIKEN, Kanagawa, Japan). These mice were backcrossed to the BALB/cA mice for eight generations. *Il1b*^−/−^ and *Il1a*^−/−^*b*^−/−^ mice were produced as described^51^. *Il1r1*^−/−^ mice were obtained from Immunex Corporation. *Il17a*^−/−^*Il1rn*^−/−^ mice were generated as described^32^. Sex- and age-matched mice, usually at 8–12 weeks of age, were used for each experiment. In some experiments, newborn mice and younger (3–4 weeks old) or elder (24 weeks old) mice were also used as described in the figure legends. All mice were kept under specific pathogen-free conditions in environmentally controlled clean rooms at the Center for Experimental Medicine and Systems Biology, The Institute of Medical Science, The University of Tokyo, and Institute for Biomedical Sciences, Tokyo University of Science. All experiments were approved by the institutional ethical committees for animal experiments and the committees for gene manipulation experiments.

### Clinical assessment of arthritis

Development of arthritis was monitored by macroscopic evaluation as described previously^31^. Briefly, each paw was graded as follows: 0, no change; 1, mild swelling; 2, obvious swelling; 3, severe swelling and ankylotic changes (maximum 12 points for individual mice).

### Histological assessment of arthritis

Whole-ankle joints were fixed in 10% formalin in 0.001 M phosphate buffer (pH 7.2), decalcified in 10% EDTA and embedded in paraffin. Serial sections (4 μm) were stained with haematoxylin and eosin. Each joint was graded on a scale of 0–3: 0, normal; 1, thickening and proliferation of the synovial lining, with slight inflammatory cell infiltration; 2, grade-1 changes plus extensive synoviocyte proliferation and severe inflammatory cell infiltration; 3, grade-2 changes plus pannus formation and bone erosion. Histological score of the ankle joint was estimated from the sum grade of both ankle joints (maximum 6 points for individual mice).

### Immunofluorescence staining

For immunohistochemical analysis, hindlimbs were embedded in Super Cryoembedding Medium (Leica Microsystems Japan, Tokyo, Japan), and frozen sections (5 μm) were generated. The sections were fixed in cold acetone for 5 min and blocked with 4% bovine serum albumin (Sigma, St Louis, MO, USA) and 5% goat serum in PBS. Antibodies used were as follows: 2 μg ml^−1^ rabbit anti-mouse IL-17A polyclonal Ab (Abcam, ab9565-100, Cambridge, UK), 5 μg ml^−1^ hamster anti-mouse γδ TCR mAb (GL3, BD Pharmingen, San Diego, CA, USA), 2.5 μg ml^−1^ Alexa Fluor 488-goat anti-rabbit-IgG (Life Technologies, Carlsbad, CA, USA), and 2.5 μg ml^−1^ Cy3-goat anti-hamster IgG (Jackson ImmunoResearch, West Grove, PA, USA). Nuclei were stained with 0.5 μg ml^−1^ 4,6-diamidino-2-phenylindole. The slides were visualized on a fluorescence microscope (Keyence, Osaka, Japan) and on an Olympus FV1000 Confocal Microscope, operated by the FluoView software (Olympus, Tokyo, Japan).

### Isolation of joint-infiltrating cells

Ankle joints were cut out and digested with 2.4 mg ml^−1^ hyaluronidase (Sigma), 1 mg ml^−1^ collagenase (Sigma) in RPMI 1640 (Sigma) plus 10% foetal bovine serum (FBS) for 1 h at 37 °C. The cells were filtered through a cell strainer with a 70-μm nylon mesh (Becton Dickinson, Franklin Lakes, NJ, USA) and washed with RPMI 1640 plus 10% FBS.

### Cell transfer

Purified γδ T cells (8 × 10^5^ cells), CD4^+^ T cells (2 × 10^7^ cells) or Thy1.2^+^ T cells (2 × 10^7^ cells) from LN (axillary, brachial, inguinal, popliteal and cervical) cells were suspended in 200 μl sterile PBS and then injected intravascularly into *scid/scid* mice.

### Antibody treatment

Non-arthritic *Il1rn*^−/−^ mice (4 weeks old) received twice weekly intraperitoneal injections of 400 μg anti-TCR γδ mAb (UC7-13D5 (ref. 53), purified from hybridoma culture supernatant) or 250 μg anti-CD4 mAb (GK1.5, purified from hybridoma culture supernatant provided by Dr Nariuchi (The University of Tokyo)). Isotype-matched Armenian hamster IgG (400 μg per mouse; Innovate Research, Noida, India) was used as a control for UC7-13D5. Isotype-matched rat IgG (250 μg per mouse; Thermo, Waltham, MA, USA) was used as a control for GK1.5. A dose of 200 μg anti-CCL2 mAb (123616; R&D Systems, Minneapolis, MN, USA), 200 μg rat IgG2b isotype control (Fitzgerald, Sudbury Road Acton, MA, USA) or PBS alone was intraperitoneally injected into non-arthritic *Il17*^*g/g*^*Il1rn*^−/−^ mice at ages of 21, 24, 27 and 30 days.

### Cell isolation

To examine LN cells by flow cytometry, axillary, brachial, inguinal and popliteal LNs were collected. To obtain FACS-purified γδ T cells, LN and/or spleen cells were first incubated with biotin-conjugated anti-mouse γδ TCR mAb (GL3; #13-5711, eBioscience, San Diego, CA, USA) (1/100 diluted) and then with MicroBeads conjugated to anti-biotin mAb (#130-090-485, Miltenyi Biotec, Bergisch Gladbach, Germany) (1/8 diluted). Labelled cells were positively selected twice using autoMACS (Miltenyi Biotec). To purify whole-T cells and CD4^+^ T cells, LN and/or spleen cells were stained with Microbeads conjugated to anti-mouse Thy1.2 mAb (#130-049-101, Miltenyi Biotec) and CD4 mAb (#130-049-201, Miltenyi Biotec), respectively (1/8 diluted), and separated using an autoMACS. MACS-purified γδ T (γδ TCR^+^CD3ɛ^+^) cells and GFP^+^ γδ T (GFP^+^ γδ TCR^+^ CD3ɛ^+^) cells were further purified using a FACSAria (Becton Dickinson). FITC-anti-mouse γδ TCR mAb (UC7-13D5; eBioscience), PE-anti-mouse γδ TCR mAb (GL3; BioLegend, San Diego, CA, USA), and APC/Cy7-anti-mouse CD3ɛ mAb (145-2C11; BioLegend) (1/100 diluted) were used for labelling. The efficiency of these cell purifications was determined by flow cytometry.

### Cell culture

For analysis of flow cytometry and concentrations of IL-17 in culture supernatants, cells from LNs and/or spleens were cultured for 72 h in RPMI 1640 containing 10% FBS with or without IL-1β (10 ng ml^−1^) (PeproTech, Rocky Hill, NJ, USA), IL-23 (10 ng ml^−1^) (R&D Systems), IL-1Ra (200 ng ml^−1^) (R&D Systems) or cytokines at the indicated concentrations (ng ml^−1^) in the absence of γδ TCR stimulation. For analysis of mRNA expression, γδ^+^CD3ɛ^+^ or γδ^−^CD3ɛ^+^ T cells from spleens were purified using a FACSAria (Becton Dickinson) and cultured for 48 h with or without IL-1β (10 ng ml^−1^) and IL-23 (10 ng ml^−1^).

### Flow cytometry

Intracellular cytokine staining was performed as described previously^54^ after stimulation with 50 ng ml^−1^ phorbol myristate acetate (Sigma), 500 ng ml^−1^ ionomycin (Sigma) and 2 μM monensin (Sigma) for 5 h. For staining of cell-surface molecules, cells were first treated with anti-mouse CD16/CD32 mAbs (2.4G2, purified from hybridoma culture supernatant) in staining buffer (HBSS containing 2% FCS and 0.1% sodium azide) to block FcR binding, and then stained with antibodies (1/100 diluted). A list of all antibodies used in the study is shown in Supplementary Table 2. For intracellular cytokine staining, cells were fixed with 4% paraformaldehyde and treated with a permeabilization buffer (0.1% saponin (Sigma) in staining buffer), and then incubated with antibodies (1/100 diluted) against intracellular cytokine. 7-Aminoactinomycin D (Sigma) was used to stain dead cells. Cells were analysed on a FACSCanto II system (Becton Dickinson), and data were analysed with FlowJo software (Tree Star).

### Measurement of cytokine

Concentrations of IL-17 in culture supernatants were determined by enzyme-linked immunosorbent assay using commercially available kits (Ready-Set-Go, eBioscience).

### RT–PCR and real-time PCR

To prepare single cells for RNA expression analysis, draining LN, mesenteric LN and spleen were digested with 200 U ml^−1^ collagenase (Sigma) for 30 min. Joints were digested with 200 Uml^−1^ collagenase (Sigma) and 2.4 mgml^−1^ hyaluronidase (Sigma). Next, these cells were filtered through a cell strainer with a 70-μm nylon mesh (Becton Dickinson). RNA from joint-, dLN-, mLN- and spleen-derived single cells and RNA from cultured cells were extracted using the GenElute Mammalian Total RNA Miniprep Kit (Sigma). Tissue total RNA from joint, draining LN, spleen, thymus, colon, lung and liver was extracted using the Sepasol reagent (Nacalai Tesque, Kyoto, Japan). All RNA were denatured in the presence of an oligo dT primer and reverse transcribed using the High Capacity cDNA Reverse Transcription Kit (Applied Biosystems, Foster City, CA, USA). Quantitative real-time PCR was performed with a SYBR Green qPCR kit (Invitrogen, Carlsbad, CA, USA) and an iCycler system (Bio-Rad, Hercules, CA, USA) with the sets of primers described in Supplementary Table 3.

### Statistics

Unless otherwise specified, all results are shown as means and s.e.m. Unpaired Student's *t*-tests were used to statistically analyse all results, except that Mann–Whitney's *U*-tests were used to evaluate disease severity and *χ*^2^-tests were used to evaluate disease incidence. Differences were considered significant at *P*<0.05.

## Additional information

**How to cite this article:** Akitsu, A. *et al*. IL-1 receptor antagonist-deficient mice develop autoimmune arthritis due to intrinsic activation of IL-17-producing CCR2^+^V^γ^6^+^γδ T cells. *Nat. Commun.* 6:7464 doi: 10.1038/ncomms8464 (2015).

## Supplementary Material

Supplementary Information Supplementary Figures 1-7, Supplementary Tables 1-3 and Supplementary Reference
