# Supplementary material for: IL-1 receptor antagonist-deficient mice develop autoimmune arthritis due to intrinsic activation of IL-17-producing CCR2+Vγ6+γδ T cells
Source: Nat Commun. 2015 Jun 25;6:7464. doi: 10.1038/ncomms8464 (PMC4521288; doi:10.1038/ncomms8464)
Supplement: Supplementary Information — Supplementary Figures 1-7, Supplementary Tables 1-3 and Supplementary Reference [file ncomms8464-s1.pdf]

Supplementary Figure 1

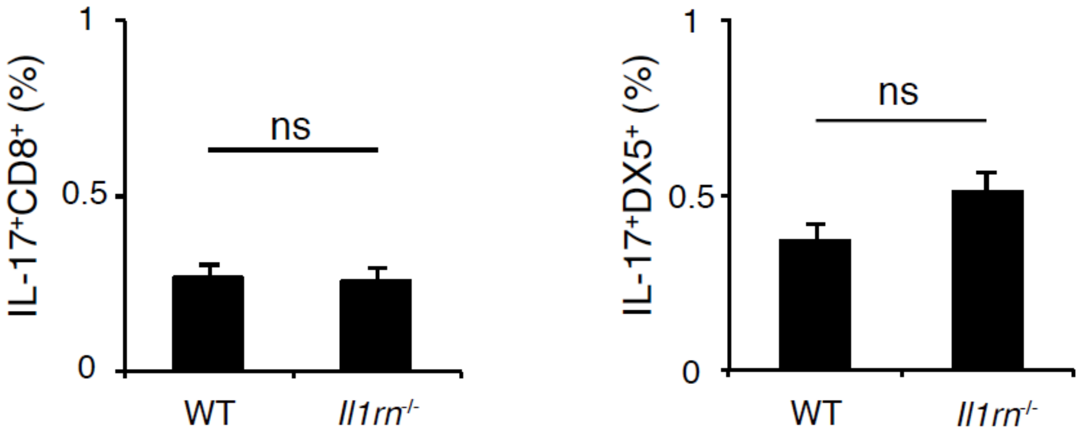

Supplementary Figure 1. Analysis of IL-17–producing cells in LNs of *Il1rn*$^{-/-}$ mice related to Figure 1

Flow cytometry of LN cells from WT (n = 3) and arthritic *Il1rn*$^{-/-}$ mice (n = 3). Cells were stimulated with P/I for 5 h, and then stained for intracellular IL-17. Quantitations of IL-17^{+}CD8^{+} (*left*) and IL-17^{+}DX5^{+} (*right*) in CD3ε^{+} cells are shown. ns, not significant vs. WT mice (unpaired Student's *t* tests). Data show means ± SEM, and are representative of two independent experiments.

Supplementary Figure 2

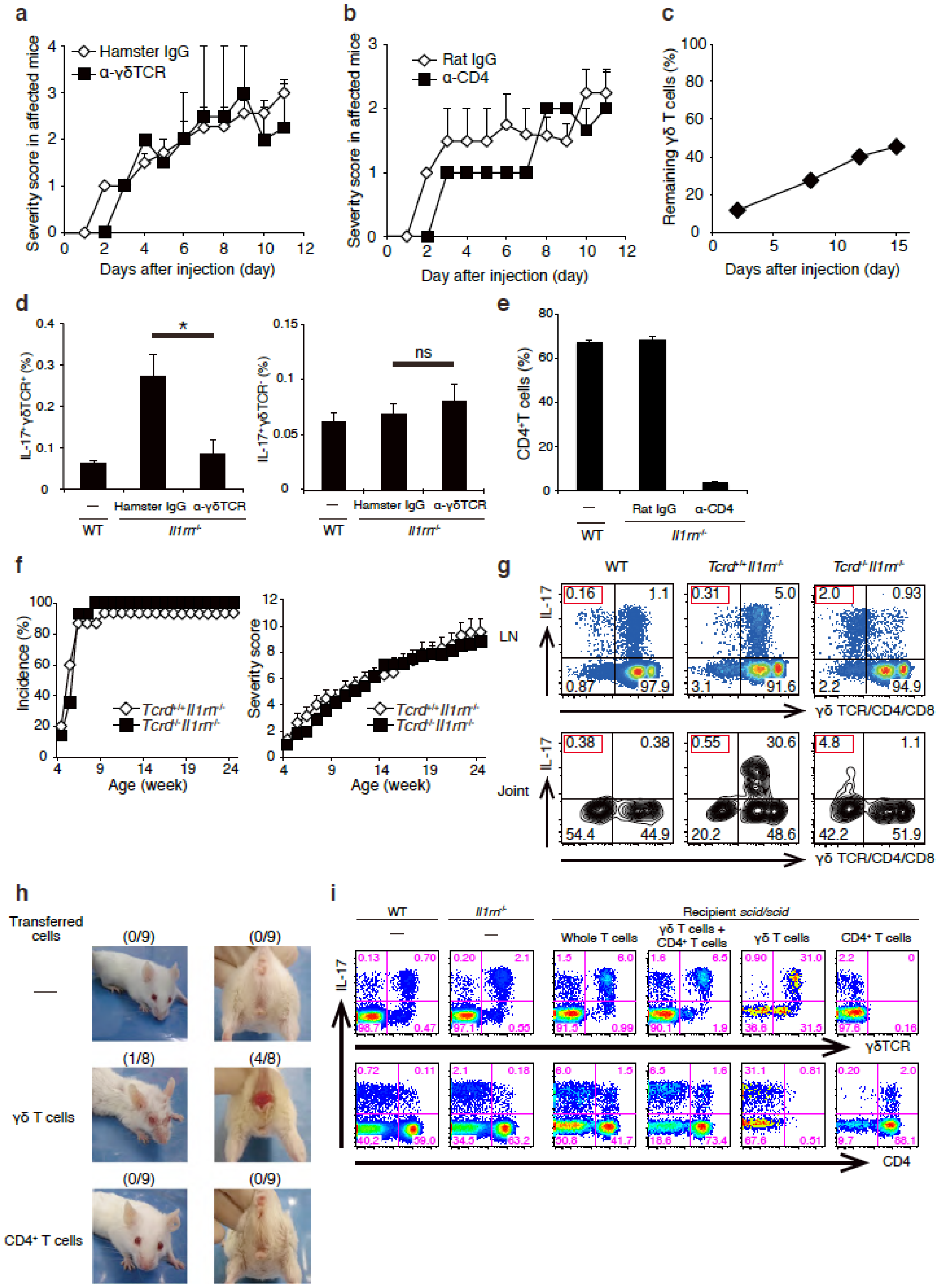

Supplementary Figure 2. Pathogenicity of γδ T cells and $CD4^+$ T cells in the development of arthritis in *Il1rn*$^{-/-}$ mice, related to Figure 2

(**a,b**) Severity scores of arthritis in affected *Il1rn*$^{-/-}$ mice after antibody treatment. Nonarthritic *Il1rn*$^{-/-}$ mice at the age of 4 weeks were injected with either anti-γδ TCR mAb (■, n = 10) or hamster IgG (◇, n = 10) (**a**), or with either anti-CD4 mAb (■, n = 10) or rat IgG (◇, n = 9) (**b**), on days 0, 3, 7, and 10 (ages of 28, 31, 35, and 38 days). Both groups are not significantly different (Mann-Whitney's *U* test). Data show means ± SEM. (**c**) Proportion of γδ T cells remaining after anti-γδ TCR antibody treatment. On days 0, 3, 7, 10, and 13, nonarthritic *Il1rn*$^{-/-}$ mice at the age of 4 weeks were injected with either anti-γδ TCR mAb or hamster IgG. On days 2, 8, 12, and 15, LN cells were collected and stained for γδ TCR. The frequency of γδ T cells among $CD3ε^+$ cells was measured by flow cytometry. Data show the average proportion of γδ T cells after treatment with anti-γδ TCR mAb (n = 3 or 4 per point) relative to the corresponding proportion after treatment with hamster IgG (n = 3 or 4 per point). Data are representative of two independent experiments. **(d)** Nonarthritic *Il1rn*$^{-/-}$ mice at the age of 20 days were injected with anti-γδ TCR mAb (n = 5) or isotype-matched hamster IgG (n = 6) every 3 days (ages of 20, 23, and 26 days), and then sacrificed at the age of 27 days. LN cells were collected from *Il1rn*$^{-/-}$ mice treated with anti-γδ TCR mAb or hamster IgG, or non-treated WT mice. Cells were stimulated with P/I for 5 h, and then analyzed for intracellular IL-17 by flow cytometry. The percentages of IL-17^+γδ TCR^+ cells (*left*) and IL-17^+γδTCR^- cells (*right*) among $CD3ε^+$ cells are shown. Data show means ± SEM. *$P < 0.05$ vs. hamster IgG-treated *Il1rn*$^{-/-}$ mice (unpaired Student's *t* test). Data are representative of two independent experiments. (**e**) Efficiency of $CD4^+$ T-cell depletion by anti-CD4 antibody treatment. The average efficiency was 94.8%. Nonarthritic *Il1rn*$^{-/-}$ mice at the age of 4 weeks received twice-weekly i.p. injection with an anti-CD4 mAb (n = 10) or rat IgG (n = 9). On day 11 after the first mAb treatment, LN cells were collected and stained for surface CD4. Data refer to the average proportions of $CD4^+$ T cells among $CD3ε^+$ cells in 9–10 mice. Age-matched and non-treated WT mice were used as controls. (**f**) Incidence (*left*) and severity score of arthritis (*right*) in affected *Tcrd*$^{+/+}$*Il1rn*$^{-/-}$ mice (◇, n=15) and *Tcrd*$^{-/-}$*Il1rn*$^{-/-}$ mice (■, n=14). Data show means ± SEM. Data are representative of two independent experiments. Both groups are not significantly different in incidence ($χ^2$ tests) or severity scores (Mann–Whitney's *U* tests). (**g**) Intracellular IL-17 expression in LN cells (*top*) and joint-infiltrating cells (*bottom*) of WT, *Tcrd*$^{+/+}$*Il1rn*$^{-/-}$, and *Tcrd*$^{-/-}$*Il1rn*$^{-/-}$ mice at the ages of 24 weeks. Cells were stimulated with P/I for 5 h, and then gated on $CD3ε^+$ cells. Numbers of upper left indicate IL-17-producing γδ $TCR^-CD4^-CD8^-$ T cells. γδTCR/CD4/CD8 refers to cells that were stained by a mixture of anti-γδTCR mAb, anti-CD4 mAb, and anti-CD8 mAb. Data are representative of 4–5 mice from two independent experiments. (**h**) *scid/scid* mice 18 weeks after transfer with γδ T cells from *Cd4*$^{-/-}$*Il1rn*$^{-/-}$ mice (n=8), $CD4^+$ T cells from *Tcrd*$^{-/-}$*Il1rn*$^{-/-}$ mice (n=9). Development of

dermatitis (*left*) and colitis (*right*) were inspected in these mice or age–matched non-treated *scid/scid* mice. Numbers in parentheses represent number of diseased mice / total number mice in the group. Data are a pool of two independent experiments. (**i**) Intracellular IL-17 expression in γδ (*top*) and $CD4^+$ T cells (*bottom*) in LNs of *scid/scid* mice 18 weeks after transfer with $Thy1.2^+$ T (Whole T) cells, γδ T cells plus $CD4^+$ T cells, $CD4^+$ T cells, or γδ T cells. Age-matched and non-treated WT and *Il1rn*$^{-/-}$ mice were used as controls. LN cells were collected and stimulated with P/I for 5 h, and then gated on $CD3\varepsilon^+$ cells. Data are representative of 4–8 mice from two independent experiments.

Supplementary Figure 3

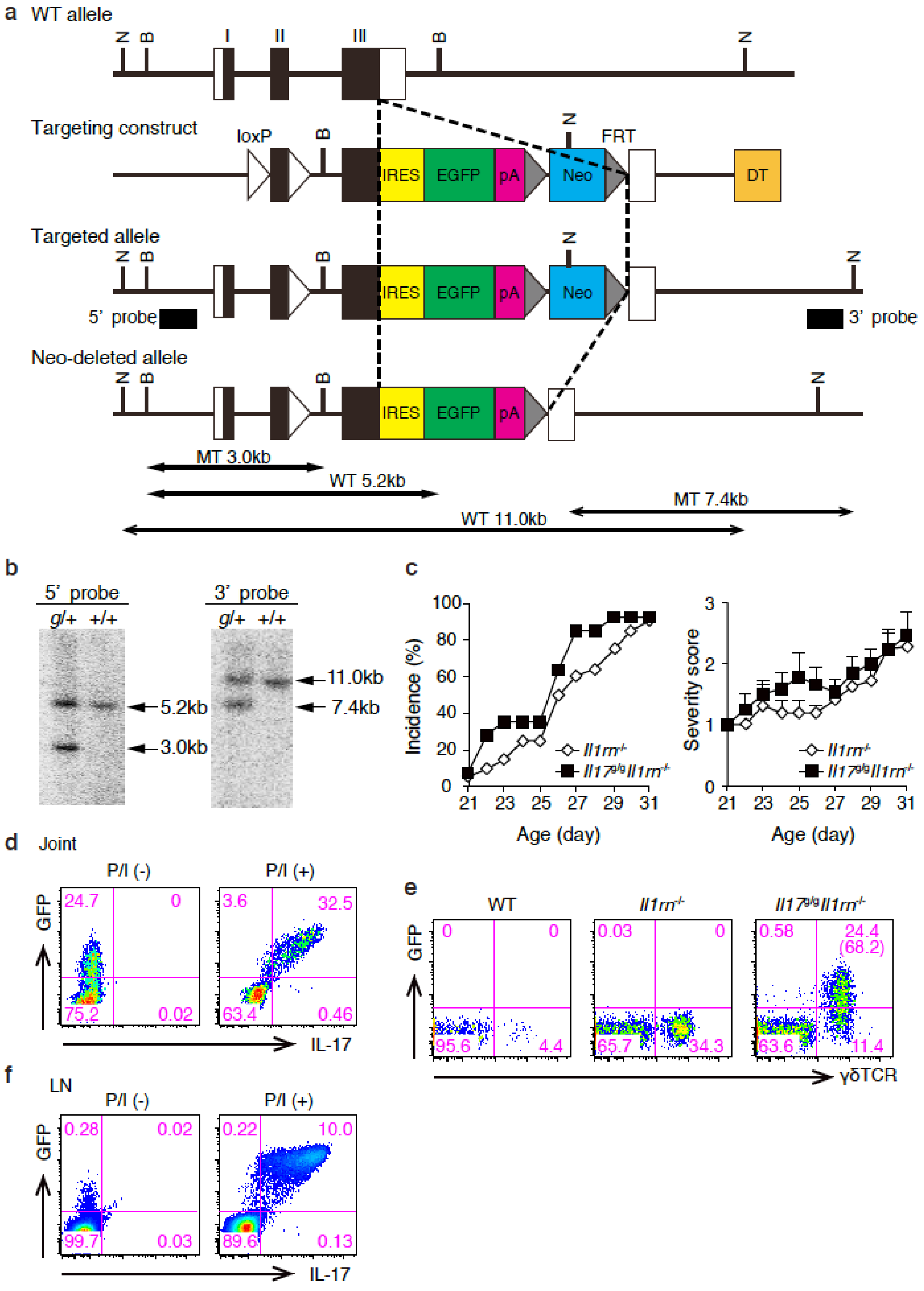

Supplementary Figure 3. Generation of *Il17*$^{g/g}$ and *Il17*$^{g/g}$*Il1rn*$^{-/-}$ mice

(**a**) Targeting strategy for generation of *Il17*$^{g/g}$ mice. *Il17*$^{g/g}$ mice were generated by homologous recombination in E14.1 ES cells. A fragment containing IRES (internal ribosome entry site)-EGFP-pA (polyadenylation signal) with the neomycin (Neo) resistance gene was inserted into just after the translational stop codon of the *Il17a* gene. For negative selection, and the diphtheria toxin A (DT) gene under the control of the MC1 promoter was attached to the 3' end of the genomic fragment. The structure of the mouse *Il17a* locus (WT allele), *Il17*$^{g/g}$ targeting construct (Targeting construct), predicted mutant *Il17a* gene after homologous recombination (Targeted allele), and predicted mutant *Il17a* gene after Flp recombinase expression vector injection (Neo-deleted allele) are shown. Filled and open boxes represented exons and UTRs, respectively. The external homologous regions shown in the targeting allele were used as genomic probes for Southern blot analysis to screen targeted clones. (**b**) Southern blot analysis was carried out using *Nco*I (N) with 5' probes and *Bam*HI (B) with 3' probes. Endogenous (5.2 kb) and targeted (3.0 kb) bands were detected using the 5' probe (*left*), and endogenous (11.0 kb) and targeted (7.4 kb) bands were detected using the 3' probe (*right*). (**c**) Incidence (*left*) and severity score of arthritis in *Il1rn*$^{-/-}$ mice (*right*) (◇, n = 20) and *Il17*$^{g/g}$*Il1rn*$^{-/-}$ mice (■, n = 14). Both groups are not significantly different in incidence (χ^2 test) or severity scores (Mann-Whitney's *U* test). Data show means ± SEM. (**d**) Flow cytometry of CD3ε$^+$ cells in the joints of *Il17*$^{g/g}$*Il1rn*$^{-/-}$ mice. Joint-infiltrating cells were treated with monensin for 5 h with (+) or without (-) PMA/ionomycin (P/I). P/I-stimulated cells were stained for intracellular IL-17 (*right*). (**e**) Flow cytometry of joint-infiltrating CD3ε$^+$ cells from WT, *Il1rn*$^{-/-}$, and *Il17*$^{g/g}$*Il1rn*$^{-/-}$ mice. Numbers in parenthesis represent percentages in CD3ε$^+$γδ TCR$^+$ cells. (**f**) Flow cytometry of CD3ε$^+$cells in LNs of *Il17*$^{g/g}$*Il1rn*$^{-/-}$ mice. LN cells were treated as shown in (**d**). All data except (**a,b**) are representative of two independent experiments.

Supplementary Figure 4

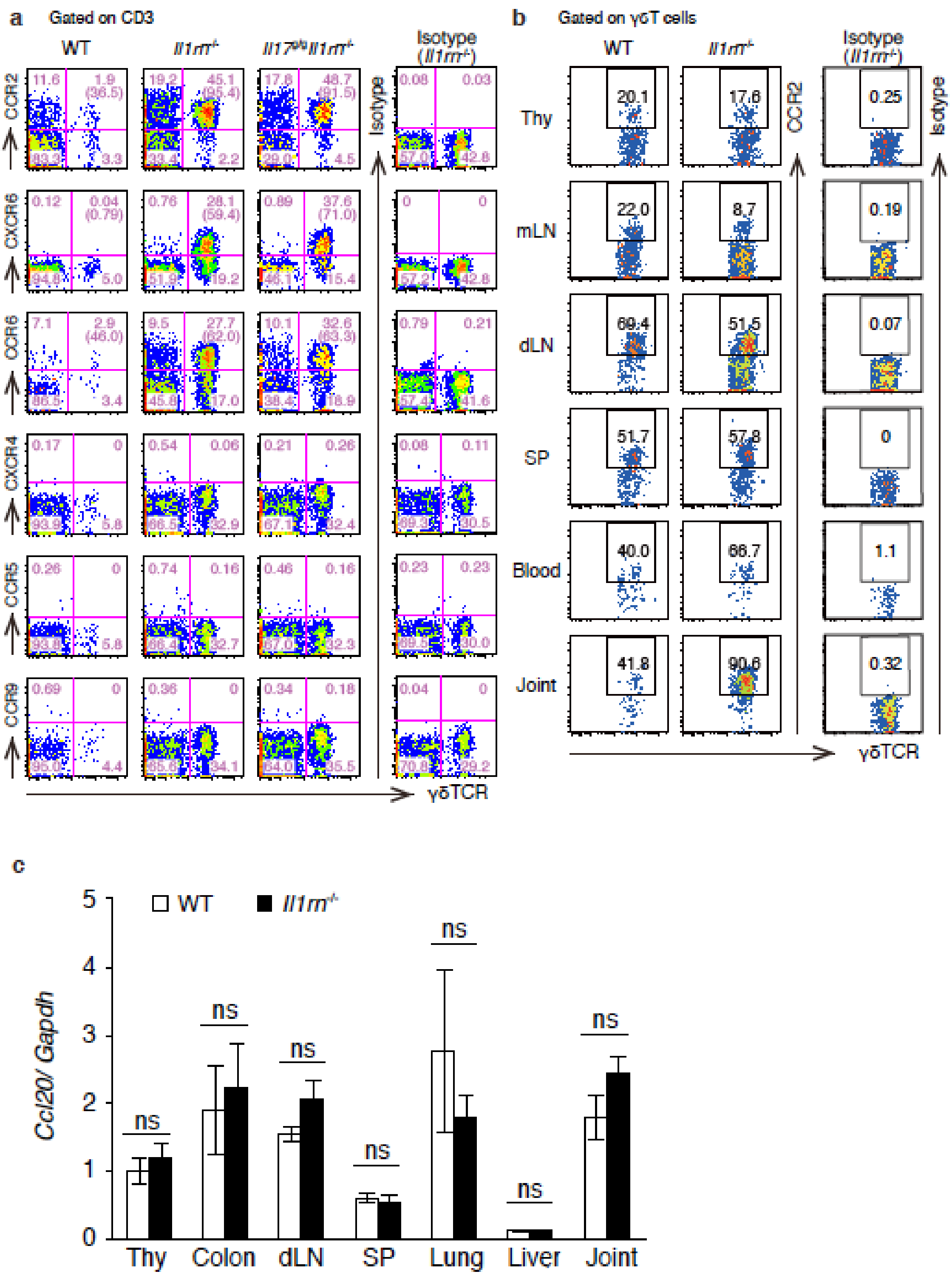

Supplementary Figure 4. Chemokine and chemokine receptor expression in *Il1rn*$^{-/-}$ mice, related to Figure 3

(**a**) Flow cytometry of chemokine receptors and γδ TCR in joint-infiltrating CD3ε^{+} cells from WT, *Il1rn*$^{-/-}$, and *Il17*$^{g/g}$*Il1rn*$^{-/-}$ mice at 16 weeks of age. Numbers refer to percentages in CD3ε^{+} cells. Data are representative of two independent experiments. (**b**) Flow cytometry of CCR2^{+} cells in CD3ε^{+}γδ TCR^{+} cells in the thymus (Thy), mesenteric LNs (mLN), draining LNs (dLN), spleens (SP), blood, and joints from WT (n=3) and *Il1rn*$^{-/-}$ (n=3) mice at 14 weeks of age. Data are representative of two independent experiments. (**c**) Levels of CCL20 mRNA were measured by quantitative PCR analysis in thymus (Thy), mesenteric LNs (mLN), draining LNs (dLN), spleens (SP), lung, liver, and joints from WT and *Il1rn*$^{-/-}$ mice. Data show means ± SEM. ns, not significant vs. WT mice (unpaired Student's *t* test). All data are representative of two independent experiments.

Supplementary Figure 5

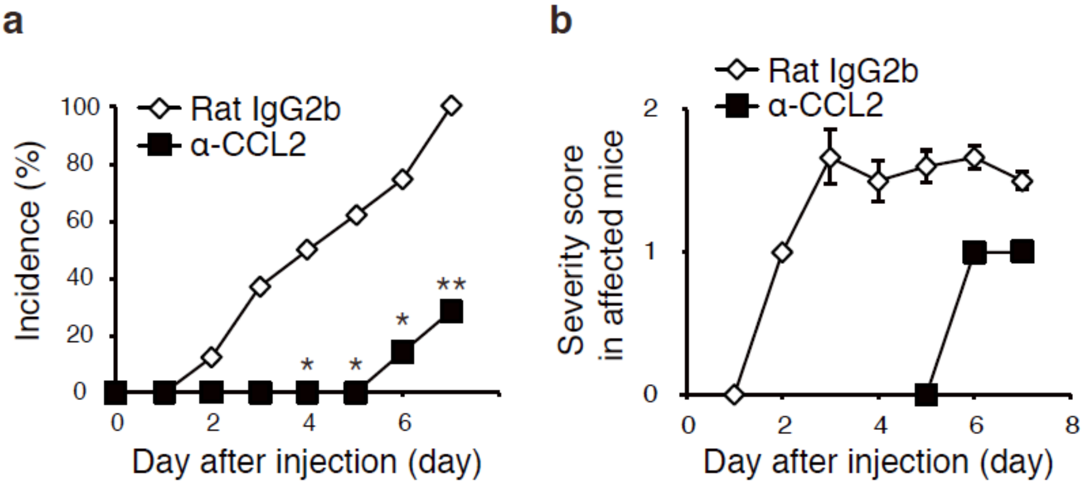

Supplementary Figure 5. Development of arthritis in *Il17*$^{g/g}$*Il1rn*$^{-/-}$ mice with anti-CCL2mAb or rat IgG2b Isotype control, related to Figure 4

Incidence (**a**) and severity scores of arthritis in total (**b**) and in affected (**c**) *Il17*$^{g/g}$*Il1rn*$^{-/-}$ mice with anti-CCL2 mAb treatment. Non-arthritic *Il17*$^{g/g}$*Il1rn*$^{-/-}$ mice at the age of 21 days were injected with anti-CCL2 mAb (■, n = 8) or rat IgG2b isotype control (◇, n = 7) every 3 days (ages of 21, 24, and 27 days). *$P < 0.05$; ** $P < 0.01$ (vs. treatment with rat IgG2b isotype control) (χ^2 test). Severity score is not significantly different **(**Mann-Whitney's *U* test**).** Data are representative of two independent experiments.

Supplementary Figure 6

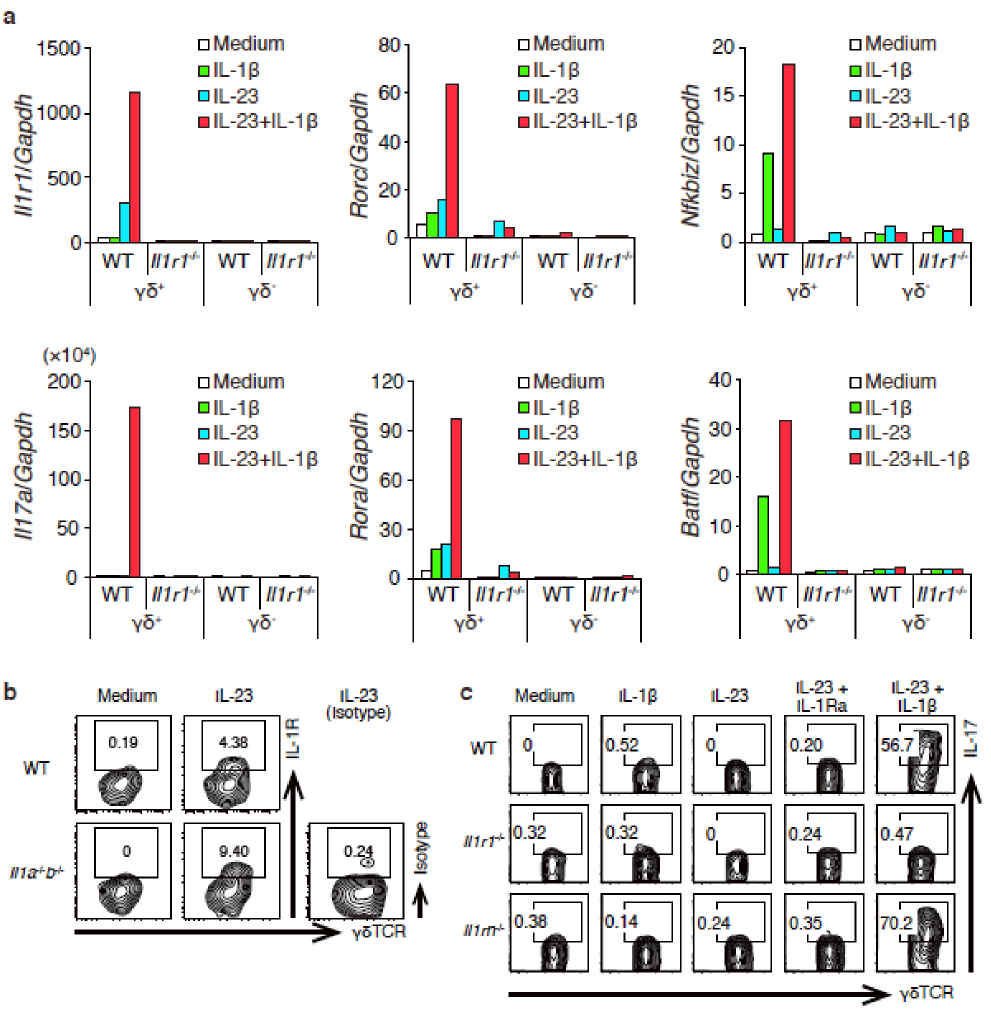

Supplementary Figure 6. IL-17 induction mechanism in γδ T cells, related to Figure 5

(**a**) Quantitative PCR analysis of mRNA levels for RORγt (*Rorc*), RORα (*Rora*), IκBζ (*Nfkbiz*), BATF (*Batf*), IL-1R (*Il1r1*), and IL-17 (*Il17a*) in FACS-purified γδ TCR^{+}CD3ε^{+} cells (γδ^{+}) or γδ TCR^{-}CD3ε^{+} (γδ^{-}) cells from spleens of WT and *Il1r1*$^{-/-}$ mice after stimulation with the indicated cytokines for 48 h. Values are shown relative to those of γδ TCR^{-}CD3ε^{+} cells from WT mice stimulated with medium only. (**b**) FACS-purified γδ T cells from spleens of WT, *Il1a*$^{-/-}$*b*$^{-/-}$, or *Il1rn*$^{-/-}$ mice were stimulated with medium only or IL-23. Flow cytometry of γδ T cells stained for surface IL-23R are shown. (**c**) FACS-purified γδ T cells from spleens of WT, *Il1r1*$^{-/-}$, and *Il1rn*$^{-/-}$ mice were stimulated with the indicated cytokines without γδ TCR stimulation for 3 days. Cells were treated with monensin for the last 5 h. After treatment, γδ T cells were stained for intracellular IL-17 and surface γδ TCR. Percentages of IL-17^{+} cells among γδ T cells are shown. Data are the representative of triplicate cultures (**b, c**). All data are representative of two independent experiments.

Supplementary Figure 7

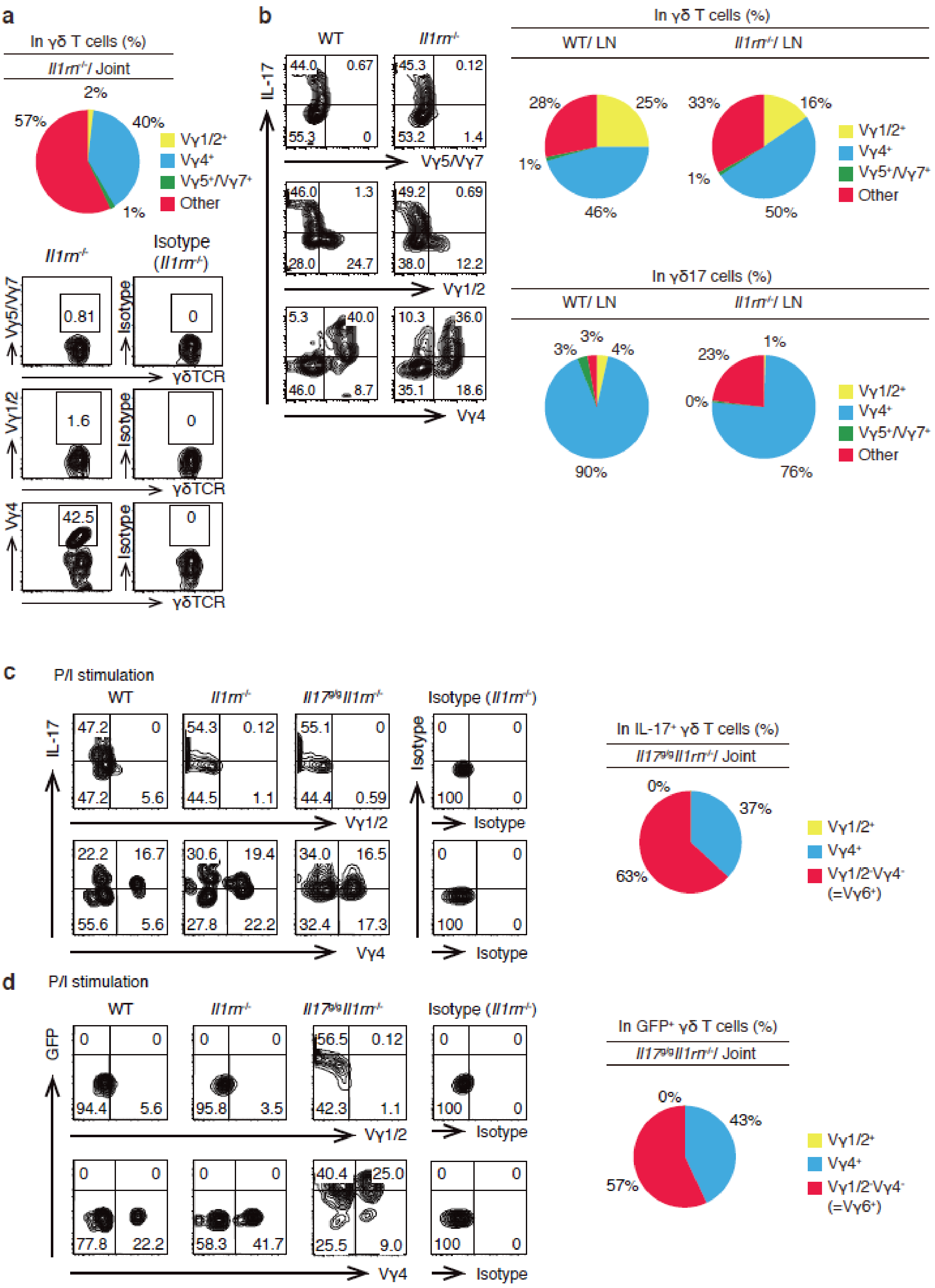

Supplementary Figure 7. Analysis of Vγ subsets in γδ17 cells of *Il17$^{g/g}$Il1rn$^{-/-}$* mice, related to Figure 6

(**a**) Proportions of Vγ subsets in 16-week-old mice were analyzed using Vγ TCR–specific antibodies except for Vγ6. Vγ1/2^{+} refers to cells that were stained by anti-Vγ1/2 mAb, which recognizes Vγ1 and/or Vγ2 TCR. Vγ5/Vγ7^{+} refers to cells that were stained by a mixture of anti-Vγ5 TCR mAb and anti-Vγ7 TCR mAbs. "Other" refers to cells that were not stained by any of those anti-Vγ TCR antibodies, i.e., Vγ1/2^{-}Vγ4^{-}Vγ5^{-}Vγ7^{-} cells. Numbers in pie charts show the average proportions of Vγ subsets among γδ T cells from *Il1rn$^{-/-}$* mouse joints (*top*). Numbers refer to percentages in CD3ε^{+}γδ TCR^{+} cells (*bottom*). We did not determine γδ subset in WT joints. (**b**) Intracellular IL-17 and Vγ TCR expression in the LNs of *Il1rn$^{-/-}$* mice. Numbers refer to percentages in CD3ε^{+}γδ TCR^{+} cells (**b**, *left*). Numbers in pie charts show averages of the indicated Vγ subsets in γδ TCR^{+}CD3ε^{+} (**b**, *upper right*) and IL-17^{+}γδ TCR^{+}CD3ε^{+} cells (**b**, *lower right*) in WT and *Il1rn$^{-/-}$* mice. (**c, d**) Intracellular IL-17 (**c**) and GFP (**d**) expressions in joint-infiltrating γδ T cells from WT, *Il1rn$^{-/-}$*, and *Il17$^{g/g}$Il1rn$^{-/-}$* mice after P/I stimulation for 5 h. Numbers refer to percentages in CD3ε^{+}γδ TCR^{+} cells (*left*). Numbers in pie charts show the percentages of Vγ6^{+} (Vγ1/2^{-}Vγ4^{-}), Vγ1/2^{+}, and Vγ4^{+} cells among IL-17^{+}γδ TCR^{+}CD3ε^{+} cells (**c**) or GFP^{+}γδ TCR^{+}CD3ε^{+} cells (**d**) (*right*). All data in pie charts are averages of three mice and representative of two independent experiments.

Supplementary Table 1. IL-1β–dependent development of arthritis in *Il1rn*$^{-/-}$ mice, related to Figure 7

Genotype	Incidence (%)	Severity score
Il1b$^{+/+}$	19/19 (100)	9.5±0.5
Il1b$^{+/-}$	13/26 (50)	4.6±0.9
Il1b$^{-/-}$	0/10 (0)	0.0±0.0

Il1rn$^{-/-}$ mice were intercrossed with *Il1b*$^{-/-}$ mice, and the development of arthritis was examined at 24 weeks of age. Disease incidence (number of diseased mice / total number mice in the group) and severity score (means ± SEM of scores in diseased mice) are shown.

Supplementary Table 2. Antibodies used for flow cytometry

Antigen	Clone	Conjugate	Source	Catalog No.
CCR2	475301	PE	R&D Systems	FAB5538P
CCR2	475301	APC	R&D Systems	FAB5538A
CCR5	HM-CCR5	PE	BioLegend	107005
CCR6	29-2L17	Brilliant Violet 421	BioLegend	129817
CCR6	29-2L17	PE	BioLegend	129803
CCR9	CW-1.2	Alexa Fluor 647	BioLegend	128707
CD121a (IL-1R)	JAMA-147	PE	BioLegend	113505
CD121a (IL-1R)	JAMA-147	APC	BioLegend	113509
CD3ε	145-2C11	APC	BioLegend	100312
CD3ε	145-2C11	APC/Cy7	BioLegend	100330
CD4	GK1.5	PE	BioLegend	100408
CD4	GK1.5	PE/Cy7	BioLegend	100422
CD4	GK1.5	APC	BioLegend	100412
CD49b (DX5)	DX5	APC	BioLegend	108909
CD8a	53-6.7	PE	BioLegend	100708
CD8a	53-6.7	APC	BioLegend	100712
CXCR4	TG12/CXCR4	Alexa Fluor 647	BioLegend	129201
CXCR6	221002	APC	R&D Systems	FAB2145A
IL-17A	TC11-18H10	Pacific Blue	BioLegend	506918
IL-23R	753317	PE	R&D Systems	FAB16861P
TCR γδ	UC7-13D5	FITC	eBioscience	11-5811
TCR γδ	GL3	PE	BioLegend	118108
TCR γδ	GL3	PE/Cy7	BioLegend	118124
TCR γδ	GL3	APC	BioLegend	118115
TCR Vγ1.1+Vγ1.2 (Vγ1+Vγ2)	4B2.9	PE	BioLegend	142703
TCR Vγ2 (Vγ4)	UC3-10A6	APC	BioLegend	137708
TCR Vγ2 (Vγ4)	UC3-10A6	FITC	BD Pharmingen	553226
TCR Vγ3 (Vγ5)	536	FITC	BD Pharmingen	553229
TCR Vγ7	F4.67	FITC	Pereira et al.[1]	

Parentheses show the alternative name used in this study

Supplementary Table 3. RT-PCR and real-time PCR primers used, related to Supplemental Experimental Procedures

Gene symbol	Forward	Reverse
RT-PCR		
Vg1	CCGGCAAAAAGCAAAAAAGT	AAGGAGACAAAGGTAGGTCCCAGC
Vg2	TTGGTACCGGCAAAAAACAAATCA	CAATACACCCTTATGACATCG
Vg4	CTTGCAACCCCTACCCATAT	AAGGAGACAAAGGTAGGTCCCAGC
Vg5	GAGGATCCCGCTTGGAAATGGATGAGA	AAGGAGACAAAGGTAGGTCCCAGC
Vg6	GATCCAAGAGGAAAGGAAAGACGGC	AAGGAGACAAAGGTAGGTCCCAGC
Vg7	GATCCAACTTCGTCAGTTCCACAAC	AAGGAGACAAAGGTAGGTCCCAGC
Vd1/Cd	ATTCAGAAGGCAACAATGAAAG	CGAATTCCACAATCTTCTTG
Vd2/Cd	AGTTCCCTGCAGATCCAAGC	CGAATTCCACAATCTTCTTG
Vd3/Cd	TTCCTGGCTATTGCCTCTGAC	CGAATTCCACAATCTTCTTG
Vd4/Cd	CCGCTTCTCTGTGAACTTCC	CGAATTCCACAATCTTCTTG
Vd5/Cd	CAGATCCTTCCAGTTCATCC	CGAATTCCACAATCTTCTTG
Vd6/Cd	TCAAGTCCATCAGCCTTGTC	CGAATTCCACAATCTTCTTG
Vd7/Cd	CGCAGAGCTGCAGTGTAACT	CGAATTCCACAATCTTCTTG
Vd8/Cd	AAGGAAGATGGACGATTCAC	CGAATTCCACAATCTTCTTG
Actb	CTAGGCACCAGGGTGTGATGG	TCTCTTTGATGTCACGCACGA
Real-time PCR		
Gapdh	TTCACCACCATGGAGAAGGC	GGCATGGACTGTGGTCATGA
Il17a	CTCCAGAAGGCCCTCAGACTAC	GGGTCTTCATTGCGGTGG
Il1r1	ACCTTCCCACAGCGGCTCCACATT	TTGTCAAGAAGCAGAGGTTTACAG
Rorc	AGCAGTGTAATGTGGCCTAC	GCACTTCTGCATGTAGACTG
Nfkbiz	CCTCCGATTTCTCCTCCACT	GTTCTTCACGCGAACACCTT
Batf	CCAGAAGAGCCGACAGAGAC	GAGCTGCGTTCTGTTTCTCC
Rora	CCCCTACTGTTCCTTCACCA	CCAGGTGGGATTTGGATATG
Ccl2	TTAAAAACCTGGATCGGAACCAA	GCATTAGCTTCAGATTTACGGGT
Cxcl16	CCTTGTCTCTTGCGTTCTTCC	TCCAAAGTACCCTGCGGTATC
Ccl5	GCTGCTTTGCCTACCTCTCC	TCGAGTGACAAACACGACTGC
Cxcl12	TGCATCAGTGACGGTAAACCA	TTCTTCAGCCGTGCAACAATC
Ccl20	GCCTCTCGTACATACAGACGC	CCAGTTCTGCTTTGGATCAGC
Il1b	CAACCAACAAGTGATATTCTCCATG	GATCCACACTCTCCAGCTGCA
Il23p19	ATGCTGGATTGCAGAGCAGTA	ACGGGGCACATTATTTTTAGTCT

Supplementary Reference

1. Pereira, P. *et al.* Developmentally regulated and lineage-specific rearrangement of T cell receptor Valpha/delta gene segments. *Eur J Immunol* **30,** 1988–1997 (2000).
